# Supplementary material for: Identification of Freezing-Responsive microRNAs and Their Targets in Chinese Jujube by Small RNA and Degradome Sequencing
Source: Int J Mol Sci. 2026 Sep 4;27(17):7912. doi: 10.3390/ijms27177912 (PMC13566890; doi:10.3390/ijms27177912)
Supplement: Supplementary file 1 [file ijms-27-07912-s001.zip › Supplementary Material S2/novel_miR_44.pdf]

Provisional ID	:	novel_miR_44
Score total	:	122466.8
Score for star read(s)	:	3.9
Score for read counts	:	122460.1
Score for mfe	:	1.2
Score for randfold	:	1.6
Score for cons. seed	:	
Total read count	:	240212
Mature read count	:	187965
Loop read count	:	0
Star read count	:	52247

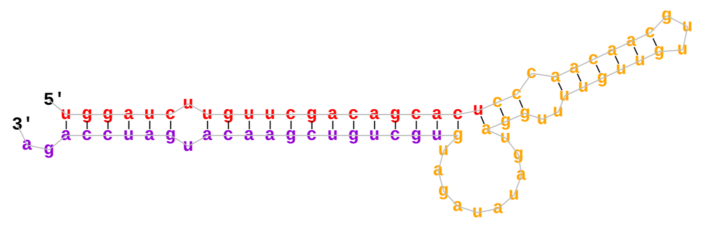

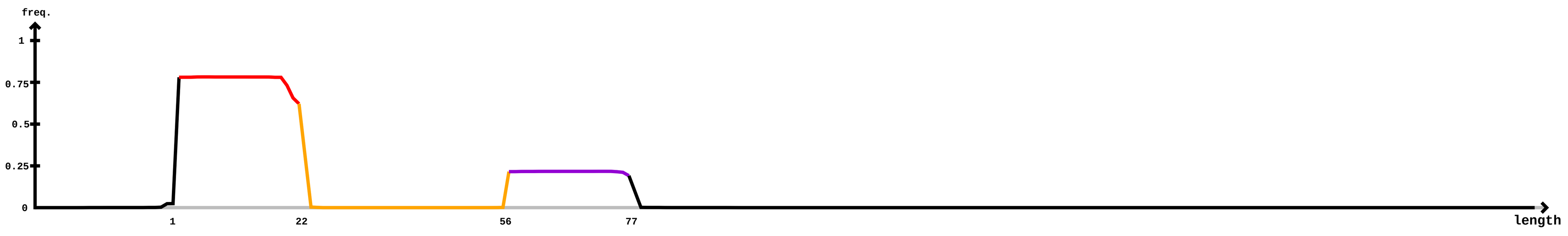

Mature Star

```
5'- cgcauguucuucguuuaccuucuggaucuuguucgacagcacucccaacaacguuguuguuuuggaugauauagaugugcugucgaacaugauccagaaggugcugucgaacaacauacaaaaaggggucuacuguacguccggcccaucaugaucuucuucuucuucuucuucuucuucuuccugaaagaguaucaaacaugggguuucuguuuguuucuucuucuuuuucuuuuuacaaguagauuua -3'   obs
    cgcauguucuucguuuaccuucuggaucuuguucgacagcacucccaacaacguuguuguuuuggaugauauagaugugcugucgaacaugauccagaaggugcugucgaacaacauacaaaaaggggucuacuguacguccggcccaucaugaucuucuucuucuucuucuucuucuucuuccugaaagaguaucaaacaugggguuucuguuuguuucuucuucuuuuucuuuuuacaaguagauuua       exp
    ...(((((.((((..((((((((((((.((((((((((((((((.((((((...))))))..)))..........)))))))))))).))))))))))))....)))).)))))..((((((((........(((...(.(((((..(((((((.(((......................))).))).))))...))))).)...))).........))))))))..................   reads   mm   sample
    ..cauguuGuucguuuaccuuc..........................   2    1   LT-3
    ...auguuGuucguuuaccuucug........................   3    1   JCK-1
    ...auguuGuucguuuaccuucug........................   2    1   JCK-2
    ....uguuGuucguuuaccuucugg.......................   2    1   JCK-2
    ....uguuGuucguuuaccuucugg.......................   2    1   JCK-1
    ......uuGuucguuuaccuucuggauc....................   2    1   LT-2
    ......uuGuucguuuaccuucuggauc....................   3    1   JCK-3
    ......uuGuucguuuaccuucuggauc....................   2    1   JCK-1
    ......uuGuucguuuaccuucuggauc....................   6    1   JCK-2
    .......uGuucguuuaccuucuggauc....................   3    1   LT-2
    .......uGuucguuuaccuucuggauc....................   10   1   JCK-1
    .......uGuucguuuaccuucuggauc....................   30   1   JCK-3
    .......uGuucguuuaccuucuggauc....................   12   1   LT-3
    .......uGuucguuuaccuucuggauc....................   12   1   LT-1
    .......uGuucguuuaccuucuggauc....................   31   1   JCK-2
    ..........ucguuuaccuucuggaucuug.................   6    0   LCK-3
    ..........ucguuuaccuucuggaucuuguuc..............   4    0   JCK-1
    ..........ucguuuaccuucuggaucuuguuc..............   1    0   JCK-2
    .............uuuaccuucuggaucuuguuc..............   3    0   LT-3
    ..............uuaccuucuggaucuuguucg.............   1    0   JCK-1
    ..............uuaccuucuggaucuuguucg.............   1    0   JCK-3
    ..............uuaccuucuggaucuuguucga............   1    0   LT-1
    ................accuucuggaucuuguucgac...........   1    0   LT-2
    ................accuucuggaucuuguucgac...........   1    0   JCK-2
    ................accuucuggaucuuguucgac...........   4    0   JCK-3
    ................accuucuggaucuuguucgac...........   4    0   JCK-1
    ................accuucuggaucuuguucgaA...........   1    1   JCK-2
    ................accuucuggaucuuguucgaca..........   1    0   JCK-2
    .................ccuucuggaucuuguucgac...........   2    0   JCK-3
    .................ccuucuggaucuuguucgac...........   2    0   LT-3
    .................ccuucuggaucuuguucgac...........   2    0   JCK-1
    .................ccuucuggaucuuguucgaca..........   29   0   JCK-2
    .................ccuucuggaucuuguucgaca..........   18   0   JT-1
```

Mature Star

```
cgcauguucuucguuuaccuucuggaucuuguucgacagcacucccaacaacguuguuguuuuggaugauauagaugugcugucgaacaugauccagaaggugcugucgaacaacauacaaaaaggggucuacuguacguccggcccaucaugaucuucuucuucuucuucuucuucuucuccugaaagaguaucaaacaugggguuucuguuuguuucuucuucuuuucuuuuuacaaguagauuua
.................ccuucuggaucuuguucgaca..................................................................................................................................................................................................................  28   0   JCK-3
.................ccuucuggaucuuguucgaca..................................................................................................................................................................................................................  22   0   JCK-1
.................ccuucuggaucuuguucgaca..................................................................................................................................................................................................................  2    0   LT-1
.................ccuucuggaucuuguucgaca..................................................................................................................................................................................................................  6    0   LT-3
.................ccuucuggaucAuguucgacagc................................................................................................................................................................................................................  2    1   LT-3
..................cuucuggaucuuguucgac...................................................................................................................................................................................................................  1    0   JCK-1
..................cuucuggaucuuguucgaca..................................................................................................................................................................................................................  1    0   JCK-2
..................cuucuggaucAuguucgaca..................................................................................................................................................................................................................  1    1   JCK-1
..................cuucuggaucAuguucgaca..................................................................................................................................................................................................................  1    1   JCK-2
..................cuucuggaucuuguucgaca..................................................................................................................................................................................................................  1    0   JCK-3
..................cuucuggaucuuguucgacagc................................................................................................................................................................................................................  1    0   JCK-1
..................cuucuggaucuuguucgacagc................................................................................................................................................................................................................  1    0   JCK-3
..................cuucuggaucAuguucgacagc................................................................................................................................................................................................................  1    1   LT-3
...................uucuggaucuuguucgac...................................................................................................................................................................................................................  3    0   LCK-3
...................uucuggaucuuguucgac...................................................................................................................................................................................................................  3    0   LT-3
...................uucuggaucuuguucgaca..................................................................................................................................................................................................................  5    0   JCK-3
...................uucuggaucuuguucgaca..................................................................................................................................................................................................................  9    0   LCK-3
...................uucuggaucuuguucgaca..................................................................................................................................................................................................................  1    0   LT-2
...................uucuggaucuuguucgaca..................................................................................................................................................................................................................  9    0   JCK-1
...................uucuggaucuuguucgaca..................................................................................................................................................................................................................  2    0   LT-3
...................uucuggCucuuguucgaca..................................................................................................................................................................................................................  1    1   LCK-3
...................uucuggaucuuguucgaca..................................................................................................................................................................................................................  1    0   JCK-2
...................uucuUgaucuuguucgacagc................................................................................................................................................................................................................  1    1   JCK-2
...................uucuggaucuuguucgacaAc................................................................................................................................................................................................................  1    1   JCK-1
...................uucuggaucuuguucgacagc................................................................................................................................................................................................................  49   0   JCK-3
...................uucuggaucuugCucgacagc................................................................................................................................................................................................................  1    1   LT-1
...................uucuggaucAuguucgacagc................................................................................................................................................................................................................  1    1   LT-1
...................uucuggCucuuguucgacagc................................................................................................................................................................................................................  1    1   JCK-3
...................uucuggaucuuguucgacagc................................................................................................................................................................................................................  36   0   LT-3
...................uucuggaucuuguucgacagc................................................................................................................................................................................................................  46   0   LCK-3
...................uucuggaucuuguucgacagc................................................................................................................................................................................................................  2    0   LCK-1
...................uucuggaucAuguucgacagc................................................................................................................................................................................................................  2    1   JCK-1
...................uucuggaucuuguucgacagc................................................................................................................................................................................................................  1    0   LCK-2
...................uucuggaucAuguucgacagc................................................................................................................................................................................................................  3    1   LT-3
...................uuNuggaucuuguucgacagc................................................................................................................................................................................................................  1    1   LT-2
...................uucuggaucuuguucgacagc................................................................................................................................................................................................................  49   0   JCK-1
...................uucuAgaucuuguucgacagc................................................................................................................................................................................................................  1    1   JCK-1
...................uucuggaucAuguucgacagc................................................................................................................................................................................................................  4    1   JCK-2
...................uucuggaucuuguucgacagc................................................................................................................................................................................................................  69   0   JCK-2
...................uucuggaucuuguucgacagc................................................................................................................................................................................................................  23   0   LT-1
...................uucuggaucAuguucgacagc................................................................................................................................................................................................................  2    1   JCK-3
...................uucuggaucuuguucgacagc................................................................................................................................................................................................................  18   0   LT-2
...................uucuggaucuuguucgacagca...............................................................................................................................................................................................................  1    0   JCK-3
...................uucuggaucuuguucgacagca...............................................................................................................................................................................................................  1    0   JCK-1
...................uucuggaucuuguucgacagca...............................................................................................................................................................................................................  3    0   JCK-2
...................uucuggaucuuguucgacagcU...............................................................................................................................................................................................................  1    1   JCK-3
...................uucuggaucuuguucgacagcU...............................................................................................................................................................................................................  2    1   JCK-1
...................uucuggaucuuguucgacagcU...............................................................................................................................................................................................................  3    1   LT-2
...................uucuggaucuuguucgacagcU...............................................................................................................................................................................................................  2    1   LT-3
...................Aucuggaucuuguucgacagca...............................................................................................................................................................................................................  2    1   JCK-3
...................uucuggaucuuguucgCcagcacu.............................................................................................................................................................................................................  1    1   JCK-2
...................uucuggaucuuguucgacagcacu.............................................................................................................................................................................................................  1    0   JCK-2
...................uucuggaucuuguucgacagcacu.............................................................................................................................................................................................................  1    0   JCK-3
....................ucuggaucuuguucgaca..................................................................................................................................................................................................................  37   0   LT-2
....................ucuggaucuuguucgacU..................................................................................................................................................................................................................  2    1   LT-1
....................ucuggaucuuguucgaca..................................................................................................................................................................................................................  22   0   JCK-3
....................ucuggaucuuguucgaca..................................................................................................................................................................................................................  53   0   LT-3
....................ucuggaucuuguucgaca..................................................................................................................................................................................................................  76   0   LCK-3
....................ucuggaucuuguucgacU..................................................................................................................................................................................................................  1    1   LT-3
....................ucuggaucuuguucgaca..................................................................................................................................................................................................................  23   0   JCK-2
....................ucuggaucuuguucgacU..................................................................................................................................................................................................................  2    1   LCK-3
....................ucuggaucuuguucgacU..................................................................................................................................................................................................................  1    1   JCK-1
....................ucuggaucuuguucgCca..................................................................................................................................................................................................................  1    1   LT-3
....................ucuggaucuuguucgaca..................................................................................................................................................................................................................  17   0   JT-3
....................ucuggaucuuguucgaca..................................................................................................................................................................................................................  17   0   LT-1
....................ucuggaucuuguucgaca..................................................................................................................................................................................................................  12   0   JT-1
....................ucuggaucuuguucgaca..................................................................................................................................................................................................................  25   0   JCK-1
....................ucuggaucuuguucgaca..................................................................................................................................................................................................................  14   0   JT-2
....................ucuggaucuuguucgacU..................................................................................................................................................................................................................  1    1   JCK-2
....................ucuggaucuuguucgacag.................................................................................................................................................................................................................  1    0   JCK-1
```

Mature **Star**

```
cgcauguucuucguuuaccuucuggaucuuguucgacagcacucccaacaacguuguuguuuuggaugauauagaugugcugucgaacaugauccagaaggugcugucgaacaacauacaaaaaggggucuacuguacguccggcccaucaugaucuucuucuucuucuucuucuucuuccugaaagaguaucaaacaugggguuucuguuuguuucuucuucuuuuucuuuuuacaaguagauuua
```

....................ucuggaucuuguucgacaU	2	1	JCK-2
....................ucuggaucuuguucgacaA	1	1	LT-1
....................ucuggaucuuguucgacag	1	0	LT-3
....................ucuggaucuuguucgacaU	2	1	JCK-3
....................ucuggaucuuguucgacagc	62	0	LCK-3
....................ucuggaucuuguucgacagc	20	0	LCK-1
....................ucuggaucuuguucgacagc	4	0	JT-3
....................ucuggaucuuguucgacagc	28	0	LCK-2
....................ucuggaucuuguucgacagc	80	0	LT-3
....................ucuggaucuuguucgacagc	69	0	LT-2
....................ucuggNucuuguucgacagc	1	1	LCK-1
....................ucuggaucuuguucgacagc	52	0	LT-1
....................ucuggCucuuguucgacagc	1	1	LT-1
....................ucuggaucuuguucgacagc	56	0	JCK-2
....................ucuggaucuuguucgacagU	1	1	LT-1
....................ucuggaucuuguucgacagc	33	0	JCK-1
....................ucuggaucuuguucgacagc	29	0	JCK-3
....................ucuggaucuuguucgacagc	5	0	JT-2
....................ucuggaucuuguucgacagcU	3	1	JCK-3
....................ucuggaucuuguucgacagca	56	0	JT-1
....................ucuggaucuugNucgacagca	2	1	JCK-1
....................uUuggaucuuguucgacagca	5	1	JCK-2
....................ucuggaucuuguucgacagca	1463	0	JCK-2
....................ucuggCucuuguucgacagca	4	1	JCK-2
....................ucuggaucuuguucgCcagca	1	1	JCK-1
....................uUuggaucuuguucgacagca	5	1	JCK-3
....................ucuggaucuuguucAacagca	1	1	JCK-1
....................ucugAaucuuguucgacagca	1	1	JCK-1
....................ucuggCucuuguucgacagca	1	1	JCK-3
....................ucuggaucuuguucgCcagca	1	1	JT-1
....................ucuggaucuuguucgacagcU	3	1	LT-2
....................ucuggaucuuguucgacagca	31	0	LCK-2
....................ucuggaucuuguucgacagca	128	0	LT-3
....................ucuggaucuuguucgacaAca	1	1	JCK-2
....................ucuggaucuuguucgacagca	9	0	JT-2
....................Ccuggaucuuguucgacagca	1	1	JCK-2
....................ucuggaucuuguucgacaUca	1	1	JCK-1
....................ucuggaucuuguucgacagca	19	0	JT-3
....................ucuggaucuuguucgacagca	27	0	LCK-1
....................ucuggaucuuguucgacagcC	1	1	JCK-1
....................ucuggaucuuguucgacagcU	8	1	JCK-2
....................ucuggaucuuguucgacagca	76	0	LCK-3
....................Ccuggaucuuguucgacagca	1	1	LT-3
....................ucNggaucuuguucgacagca	1	1	LCK-1
....................ucuggaucCuguucgacagca	1	1	LT-2
....................ucuggaucuuguucgacagca	882	0	JCK-1
....................ucuggaucuuguucgacagcU	2	1	LT-3
....................ucCggaucuuguucgacagca	1	1	JCK-3
....................ucuggaucuuguucgacagca	109	0	LT-1
....................ucuggaucCuguucgacagca	1	1	JCK-2
....................ucuggaucuuguucgacagcN	1	1	JCK-1
....................ucuggaucuuguucgacagUa	1	1	JCK-3
....................uNuggaucuuguucgacagca	2	1	JCK-2
....................ucuggaucuuguucgaUagca	1	1	JCK-3
....................uUuggaucuuguucgacagca	5	1	JCK-1
....................ucuggaucuuguucgCcagca	1	1	LT-1
....................ucuggaucuuguucgaAagca	1	1	JCK-1
....................ucuggaucuuguucgacagca	1162	0	JCK-3
....................ucuggaucuuguucgacaUca	3	1	JCK-2
....................ucuggaucuuguucgacagcU	2	1	JCK-1
....................ucuggaucuuguucgaAagca	1	1	JCK-2
....................ucuggaucuuguucgaAagca	1	1	JCK-3
....................ucuggauAuuguucgacagca	1	1	JCK-3
....................ucuggaucuuguucgacagca	83	0	LT-2
....................ucuggaucuuguucgacagcU	3	1	LCK-3
....................ucuggaucuuguucgacagcac	30	0	JCK-3
....................ucuggaucuuguucgacagcac	11	0	LT-3
....................ucuggaucuuguucgacagcaA	1	1	JCK-3
....................ucuggaucuuguucgacagcac	16	0	LCK-3
....................ucuggaucuuguucgacagcac	37	0	JCK-2

Mature **Star**

cgcauguucuucguuuaccuucuggaucuuguucgacagcacucccaacaacguuguuguuuuggaugauauagaugugcugucgaacaugauccagaaggugcugucgaacaacauacaaaaggggucuacuguacguccggcccaucaugaucuucuucuucuucuucuucuucuucuuccugaaagaguaucaaacaugggguuucuguuuguuucuucuucuuuucuuuuuacaaguagauuua			
....................ucuggaucuuguucgacagcac	10	0	LT-1
....................ucuggaucuuguucgacagcac	7	0	LT-2
....................ucuggaucuuguucgacagcac	16	0	JCK-1
....................ucuggaucuuguucgacagcacA	1	1	JCK-3
....................ucuggaucuuguucgacagcacC	1	1	LT-3
....................ucuggaucuuguucgacagcacu	8	0	LCK-3
....................ucuggaucuuguucgacagcacu	4	0	JCK-1
....................ucuggaucuuguucgacagcacA	1	1	JCK-2
....................ucuggaucuuguucgacagcaAu	3	1	LT-3
....................ucuggaucuuguucgacagcaUu	1	1	LT-3
....................ucuggaucuuguucgacagcacA	2	1	JCK-1
....................ucuggaucuuguucgacagcacu	3	0	LT-3
....................ucuggaucuuguucgacagcacu	2	0	LT-2
....................ucuggaucuuguucgacagcacu	9	0	JCK-2
....................ucuggaucuuguucgacagcacu	5	0	JCK-3
....................ucuggaucuuguucgacagcacuU	1	1	LT-1
....................ucuggaucuuguucgacagcacuc	1	0	LT-3
....................ucuggaucuuguucgacagcacuc	3	0	JCK-2
....................ucuggaucuuguucgacagcacuc	1	0	JCK-3
....................ucuggaucuuguucgacagcacuc	3	0	LT-1
....................ucuggaucuuguucgacagcacuc	5	0	JCK-1
.....................cuggaucuuguucgacagc	3	0	LT-2
.....................cuggaucuuguucgacagc	4	0	LT-3
.....................cuggaucuuguucgacagc	1	0	LT-1
.....................cuggaucuuguucgacagc	1	0	LCK-3
.....................cuggaucuuguucgacagca	2	0	LT-1
.....................cuggaucuuguucgacagca	12	0	JCK-3
.....................cuggaucuuguucgacagca	7	0	LCK-3
.....................cuggaucuuguucgacagca	15	0	JCK-1
.....................cuggaucuuguucgacagcU	1	1	JCK-3
.....................cuggaucAuguucgacagca	1	1	LT-1
.....................cuggaucuuguucgacagca	17	0	JCK-2
.....................cuggaucuuguucgacagca	4	0	LT-3
.....................cuggaucuuguucgacagcac	2	0	JCK-2
.....................cuggaucuuguucgacagcac	1	0	JCK-3
.....................cuggaucuuguucgacagcac	4	0	LT-2
.....................cuggaucuuguucgacagcacu	3	0	LT-2
.....................cuggaucuuguucgacagcacu	2	0	LT-1
.....................cuggaucuuguucgacagcacu	27	0	JCK-2
.....................cuggaucuuguucgacagcacu	5	0	LCK-3
.....................Uuggaucuuguucgacagcacu	1	1	JCK-3
.....................cuggaucuuguucgacagcacu	13	0	JCK-3
.....................cuggaucuuguucgacagcacu	15	0	JCK-1
.....................cuggaucuuguucgacagcacu	2	0	LT-3
.....................Uuggaucuuguucgacagcacuc	1	1	JCK-2
.....................cuggaucuuguucgacagcacuc	1	0	JCK-1
.....................cuggaucuuguucgacagcacuc	2	0	LT-3
.....................cuggaucuuguucgacagcacuc	6	0	JCK-2
.....................cuggaucuuguucgacagcacuc	1	0	JCK-3
......................uggaucuuguucgCcagc	8	1	LT-2
......................ugNaucuuguucgacagc	1	1	LCK-3
......................uggaucuuguucgCcagc	2	1	LT-1
......................uggaucuuguucgacagc	74	0	JT-3
......................uggaucuuguucgGcagc	1	1	LCK-3
......................uggCucuuguucgacagc	1	1	JCK-1
......................uggCucuuguucgacagc	19	1	LT-2
......................uNgaucuuguucgacagc	1	1	JCK-2
......................uggaucuuguucgaUagc	1	1	JCK-1
......................uggaucuuguucgaUagc	1	1	LT-2
......................uggaucuuguucgacagU	2	1	JCK-2
......................uggCucuuguucgacagc	3	1	LT-1
......................uggaucuuguucgacaUc	1	1	JCK-1
......................uggaucuuguucgacagU	15	1	LT-3
......................uggaucuuguucgacagc	794	0	JCK-2
......................uggaucuuguucgacaAc	1	1	LCK-3
......................uggaucuuguucgNcagc	2	1	LCK-1
......................uggaucuuguucgCcagc	4	1	LCK-3
......................uggaucuuguucgacCgc	1	1	JCK-3
......................uAgaucuuguucgacagc	1	1	LCK-1
......................uggaucuuguucgCcagc	1	1	LT-3

Mature — Star

```
cgcauguucuucguuuaccuucuggaucuuguucgacagcacucccaacaacguuguuguuuuggaugauauagaugugcugucgaacaugauccagaaggugcugucgaacaacauacaaaaaggggucuacuguacguccggcccaucaugaucuucuucuucuucuucuucuucuuccugaaagaguaucaaacaugggguuucuguuuguuucuucuucuuuuucuuuuuacaaguagauuua
```

......................uggaucuuguucgaAagc	1	1	LT-2
......................uggaucuuguucgacagc	1173	0	LT-3
......................uggaucuuguucgacaUc	6	1	LT-2
......................ugAaucuuguucgacagc	1	1	LCK-3
......................uggGucuuguucgacagc	1	1	JT-1
......................uggCucuuguucgacagc	3	1	JCK-3
......................ugAaucuuguucgacagc	1	1	JCK-3
......................uggaucuuguucUacagc	1	1	JCK-3
......................uggaucuuguucgacagc	659	0	LCK-1
......................uAgaucuuguucgacagc	2	1	JCK-2
......................uggCucuuguucgacagc	3	1	LT-3
......................uNgaucuuguucgacagc	1	1	LCK-1
......................uggaucuuguucgacaAc	1	1	LT-1
......................uggaucuuguucgacagU	1	1	JCK-1
......................uNgaucuuguucgacagc	1	1	JT-1
......................uggCucuuguucgacagc	1	1	LCK-2
......................uggaucuuguucgacagU	1	1	LT-2
......................ugAaucuuguucgacagc	2	1	LT-2
......................uggaucuuguucgacagc	2025	0	LT-2
......................uAgaucuuguucgacagc	2	1	LCK-3
......................ugAaucuuguucgacagc	2	1	JCK-2
......................ugNaucuuguucgacagc	4	1	LT-2
......................uggaCcuuguucgacagc	4	1	LCK-3
......................uggaucuuguucgacagc	890	0	LT-1
......................uggaucuuguucgaUagc	1	1	LCK-3
......................uggaucuuguucgacaAc	1	1	LT-2
......................uggaucuuguuNgacagc	1	1	JCK-1
......................ugAaucuuguucgacagc	1	1	LCK-1
......................uggGucuuguucgacagc	2	1	JCK-1
......................uggaucuuguucgacagU	3	1	LT-1
......................uggaucuuguucgacagc	454	0	JCK-1
......................uggaucuuguucAacagc	1	1	LT-2
......................uUgaucuuguucgacagc	1	1	LT-3
......................uggaucuuguucgacagc	3342	0	LCK-3
......................ugUaucuuguucgacagc	1	1	LT-3
......................uggaucuuguucgacagU	3	1	JCK-3
......................uggaucuuguucgCcagc	1	1	LCK-1
......................uggaucuuguucgacagc	81	0	JT-1
......................uggaucuuguucgCcagc	2	1	JCK-3
......................uNgaucuuguucgacagc	1	1	LT-3
......................uggaucuuguucgacCgc	1	1	LT-2
......................uggaucuuguucgacagA	1	1	LT-3
......................ugNaucuuguucgacagc	1	1	LCK-1
......................uggaucuuguucgacagU	5	1	LCK-3
......................uggaucuuguucgacagc	663	0	LCK-2
......................uggaucuuguucgacaUc	2	1	JCK-3
......................uggCucuuguucgacagc	1	1	LCK-1
......................uggaucuuguuNgacagc	1	1	JCK-2
......................uggaucuuguucgCcagc	2	1	LCK-2
......................uggCucuuguucgacagc	6	1	LCK-3
......................uAgaucuuguucgacagc	1	1	LT-2
......................uggaucuuguucgCcagc	3	1	JCK-2
......................uggaucuuguucgacagc	48	0	JT-2
......................uggaucuuguucgacGgc	1	1	JCK-2
......................uggaucuuguucgaAagc	1	1	LCK-3
......................Cggaucuuguucgacagc	1	1	LT-2
......................uggaucuuguuNgacagc	1	1	JT-1
......................uggaucuuguucgacagc	616	0	JCK-3
......................uggaucuuguucgacaUca	1	1	JCK-3
......................uggGucuuguucgacagca	1	1	JT-2
......................uggaucuuguucgacCgca	1	1	LT-3
......................uggaucuuguucgacagca	1963	0	LCK-2
......................uggaucuuguucgacagUa	9	1	LCK-3
......................uggaucuuguucgacagUa	5	1	LT-1
......................uggaucuuguucgCcagca	1	1	LCK-3
......................uggaucuuguucgacagUa	11	1	LT-2
......................uggCucuuguucgacagca	1	1	JCK-1
......................uggaucuuguucgacagcC	1	1	JCK-2
......................uggaucuuguucgacagca	932	0	JCK-2
......................uggaucuuguucgacagUa	1	1	JCK-1

Mature **Star**

cgcauguucuucguuuaccuucuggaucuuguucgacagcacucccaacaacguuguuguuuuggaugauauagaugugcugucgaacaugauccagaaggugcugucgaacaacauacaaaaaggggucuacuguacguccggcccaucaugaucuucuucuucuucuucuucuucuuccugaaagaguaucaaacaugggguuucuguuuguuucuucuucuuuucuuuuacaaguagauuua

uggaucuuguucgacagcU	194	1	JCK-2
uNgaucuuguucgacagca	1	1	LCK-1
uggaucuuguucgaNagca	4	1	LCK-1
uggNucuuguucgacagca	1	1	JCK-1
uggaucuuguucgacagcU	75	1	LT-1
uggaucuuguucgacagcG	2	1	LT-3
ugAaucuuguucgacagca	1	1	LT-2
uggCucuuguucgacagca	2	1	LCK-1
uggGucuuguucgacagca	1	1	LCK-3
uggaucCuguucgacagca	1	1	JT-1
uggaucuuguucgacagAa	1	1	LT-3
uNgaucuuguucgacagca	3	1	JCK-2
uggaucuuguucgacagcU	62	1	LCK-2
uggaucuuguucgacagGa	1	1	JCK-1
uggCucuuguucgacagca	1	1	JCK-3
uggaucuuguucgaAagca	3	1	LCK-2
uggaucuuguucgacagcC	1	1	LCK-2
uUgaucuuguucgacagca	1	1	LCK-2
uggaucuuguucgacCgca	2	1	LT-2
uggaucuuguucgacagca	294	0	JT-3
uggaucuuguucgacCgca	1	1	LCK-3
uggaucuuguucgaUagca	1	1	LT-2
uggaucuuguucAacagca	1	1	LT-3
uggaucuuguucgacagcU	299	1	LCK-3
uggaucuuguucgacagcU	164	1	LT-3
uggGucuuguucgacagca	1	1	LCK-2
uggaucuuguucgacagca	575	0	JT-1
uggaucuuguucgaUagca	4	1	JCK-2
uNgaucuuguucgacagca	2	1	JCK-3
uggaucuuguucgacagcC	2	1	LT-3
uggaucuuguucgaGagca	1	1	LCK-1
uggaCcuuguucgacagca	2	1	JCK-3
uggaucuuguucgCcagca	2	1	JCK-3
uggaucuuguucgacagcU	162	1	JCK-3
uggaucuuguucgacagcU	68	1	LCK-1
uggaucuuguucgacagca	529	0	JCK-1
uggaucCuguucgacagca	1	1	JCK-1
uggaucuuguucgacGgca	1	1	JT-1
uAgaucuuguucgacagca	2	1	LCK-3
uggaucuuguucgCcagca	1	1	JT-1
uggaucuuguucgacagca	606	0	JCK-3
uggCucuuguucgacagca	5	1	LT-2
uggaucuuguucgCcagca	2	1	LT-1
uggaucuuguucgacaUca	3	1	JCK-2
uggaucuuguucgacagca	1113	0	LT-3
uggaucuuguucgaAagca	1	1	LT-3
uggaucuuguucgacagca	496	0	LT-1
uggaucuuguucgacaAca	1	1	JT-1
uggaucuuguucgacagcC	1	1	JT-1
uggaucuuguuNgacagca	2	1	JT-1
Cggaucuuguucgacagca	1	1	LCK-2
uggaucuuguucgaUagca	4	1	JCK-1
uggaucuuguucgacagcG	3	1	JCK-2
uggaucuuguucgacagcU	56	1	JT-1
uggaucuuguucgacaAca	1	1	LCK-1
uggaucuuguucgaAagca	1	1	LT-2
uggauNuuguucgacagca	3	1	LCK-1
uggaucuuguucgacagcU	5	1	JT-3
uggaucuuguucNacagca	1	1	LCK-1
uggaucuuguucgacagca	237	0	JT-2
uNgaucuuguucgacagca	3	1	JT-1
uggaucuuguucgCcagca	1	1	LCK-2
uggaucuuguucgCcagca	3	1	LT-2
uggCucuuguucgacagca	5	1	LT-1
ugAaucuuguucgacagca	1	1	JCK-2
uggaucuuguucgacagca	2250	0	LCK-3
uAgaucuuguucgacagca	1	1	LT-2
uggaucuuguucgCcagca	5	1	LCK-1
uggaucuuguucgacagcC	1	1	LCK-3
uggaucuuguucgacaUca	15	1	LCK-3

Mature **Star**

cgcauguucuucguuuaccuucuggaucuuguucgacagcacucccaacaacguuguuguuuuggaugauauagaugugcugucgaacaugauccagaaggugcugucgaacaacauacaaaaaggggucuacuguacguccggcccaucaugaucuucuucuucuucuucuucuucuucuuccugaaagaguaucaaacaugggguuucuguuuguuucuucuucuuuuucuuuuuacaaguagauuua			
......................uggaucuuguucgacagUa	5	1	LT-3
......................uggaucuuguucgacagca	1924	0	LCK-1
......................uggaucuuguucgacagGa	1	1	JCK-2
......................ugNaucuuguucgacagca	5	1	LT-2
......................uggGucuuguucgacagca	1	1	JT-1
......................uggaucuuguucgacaUca	1	1	LT-3
......................uggaucuuguucgacagca	1184	0	LT-2
......................uggaucuuAuucgacagca	1	1	LCK-1
......................uggCucuuguucgacagca	8	1	LCK-2
......................uggaucCuguucgacagca	1	1	LT-3
......................uggCucuuguucgacagca	1	1	LT-3
......................uggaucuuguucgacagcU	9	1	JT-2
......................uggaucuuguucgaUagca	2	1	LCK-2
......................uggaucCuguucgacagca	1	1	LCK-1
......................uggCucuuguucgacagca	2	1	LCK-3
......................uggaucuuguucgacCgca	2	1	JCK-2
......................Cggaucuuguucgacagca	1	1	LT-2
......................uggauUuuguucgacagca	1	1	LCK-3
......................uggauUuuguucgacagca	1	1	JCK-3
......................uggaucuuguuNgacagca	1	1	JT-2
......................uggaucuuguucgacagcU	211	1	LT-2
......................uggCucuuguucgacagca	1	1	JCK-2
......................uggaucuuguucgacagUa	10	1	JT-1
......................uggaucuuguucgaUagca	1	1	JCK-3
......................uUgaucuuguucgacagca	1	1	LT-3
......................uAgaucuuguucgacagca	1	1	JCK-2
......................uggaucuuguucgacCgca	2	1	LCK-2
......................uggaucuuguucgacagUa	6	1	JCK-2
......................uggaucuuguucgNcagca	6	1	LCK-1
......................uggaucuuguucgacagcU	155	1	JCK-1
......................uggaucuuguuNgacagca	5	1	JCK-1
......................uggaucuuguuGgacagca	9	1	LCK-3
......................uggaucuuguucgacaAca	1	1	LT-1
......................ugCaucuuguucgacagca	1	1	LT-2
......................uggaucuuguucgacagUa	4	1	JCK-3
......................uggCucuuguucgacagcac	2	1	LT-3
......................uggaucuuguucgacagcaA	2	1	LT-3
......................uNgaucuuguucgacagcac	1	1	JCK-1
......................uggCucuuguucgacagcac	2	1	JCK-2
......................uggaucuuguucgaUagcac	3	1	JCK-3
......................uggaucuuguucgacagcUc	1	1	LT-1
......................uggaucuuguucgaAagcac	1	1	LT-3
......................uggaucuuguucgacCgcac	1	1	LT-2
......................uggaucuuguucgacagcNc	1	1	JCK-1
......................uggaucuuguucgacagcGc	1	1	LT-2
......................uggaucuuguucgacagcac	821	0	LCK-1
......................ugUaucuuguucgacagcac	1	1	JCK-3
......................ugAaucuuguucgacagcac	2	1	JCK-1
......................uggaucuuguucgGcagcac	1	1	JCK-1
......................uggaucuuguucgacagcUc	3	1	LT-3
......................uggaucuuguucgacCgcac	2	1	JCK-1
......................uggaucuuguucgCcagcac	6	1	LCK-3
......................uggaucuuguucgacagcCc	1	1	LCK-2
......................uggaucuuguucgacGgcac	1	1	LT-2
......................uggaucuuguucgaAagcac	1	1	JCK-1
......................uggaucuuguucgacagcaU	1	1	LCK-3
......................uggaucuuguucgacagcCc	3	1	LT-2
......................uggaucuuguucgacagcac	72	0	JT-2
......................uggCucuuguucgacagcac	5	1	LCK-3
......................ugUaucuuguucgacagcac	1	1	LT-3
......................uggaucuuguucgCcagcac	1	1	JCK-1
......................uggCucuuguucgacagcac	11	1	LT-2
......................uggaCcuuguucgacagcac	1	1	LCK-3
......................uggaucuuguucgacagcac	454	0	JCK-1
......................uggaucuuguucgacagcUc	2	1	JCK-2
......................uggaucuuguucgCcagcac	1	1	LT-3
......................uggaucuuguucgaGagcac	1	1	LCK-1
......................uggaucuuguucgacagcac	1028	0	LT-2
......................uggaucuuguucgacagcaU	16	1	LT-3
......................uggaucuuguucgCcagcac	5	1	LT-2

Mature Star

cgcauguucuucguuuaccuucuggaucuuguucgacagcacucccaacaacguuguuguuuuggaugauauagaugugcugucgaacaugauccagaaggugcugucgaacaacauacaaaaggggucuacuguacguccggcccaucaugaucuucuucuucuucuucuucuucuucuuccugaaagaguaucaaacauggggguuucuguuuguuucuucuucuuuuucuuuuuacaaguagauuua

......................uggCucuuguucgacagcac	1	1	JT-3
......................uggaucuuguucgacagcaU	12	1	JCK-3
......................uggCucuuguucgacagcac	2	1	LCK-1
......................uggaucuuguucgacagcCc	1	1	JT-3
......................uggaucuuguucgacaAcac	1	1	JCK-1
......................uggaucuuguucgaNagcac	4	1	LCK-1
......................uggaucuuguuNgacagcac	1	1	JT-2
......................uggaucuuguucgacagcCc	1	1	LCK-3
......................uggaucuuguucgacagcac	164	0	JT-1
......................uggaucuuguucgacagcaU	11	1	LT-2
......................uggaucuuguucgacagcUc	3	1	LT-2
......................uggaucuuguucgaUagcac	3	1	JCK-2
......................ugAaucuuguucgacagcac	1	1	LT-2
......................ugNaucuuguucgacagcac	4	1	LT-2
......................uggauNuuguucgacagcac	2	1	LCK-1
......................uggaucuuguucgacagcac	564	0	LT-1
......................uggaucuuguucgaUagcac	1	1	JCK-1
......................uggaucuuguucgNcagcac	4	1	LCK-1
......................uggaucuuguucgacagcaU	10	1	LT-1
......................uggaucuuguucUacagcac	1	1	LT-1
......................uggaucuuguucgacagcaA	1	1	JCK-1
......................ugAaucuuguucgacagcac	1	1	LCK-3
......................uAgaucuuguucgacagcac	1	1	LCK-3
......................uggaucuuAuucgacagcac	1	1	LCK-1
......................uAgaucuuguucgacagcac	1	1	LT-3
......................uggaucuugCucgacagcac	1	1	LT-2
......................uggaucuuguucgacagcNc	1	1	LCK-1
......................uggaucuuguucgacagcac	796	0	LCK-2
......................uggaucuuguucgacagcUc	1	1	JCK-1
......................uggaucuuguucgacagcaA	3	1	JCK-2
......................uggaucuuguucgacagcaU	19	1	JCK-2
......................uggaucuuguucgGcagcac	1	1	LCK-1
......................uggCucuuguucgacagcac	4	1	LCK-2
......................uggaucuuguucgaUagcac	1	1	LT-2
......................uggaucuuguucgacagcac	508	0	JCK-3
......................uggaucuuguucgacagcac	2109	0	LCK-3
......................ugUaucuuguucgacagcac	1	1	LT-2
......................uggaucuuguucgCcagcac	1	1	LCK-2
......................uggaucuuguucgacagcac	762	0	LT-3
......................uggaucuuguucgacagcac	727	0	JCK-2
......................uggaucuuguucgacGgcac	1	1	LT-1
......................uggaucuuguucgacGgcac	1	1	JCK-2
......................uggaucuuguucgacagcaA	9	1	JCK-3
......................uggCucuuguucgacagcac	1	1	LT-1
......................uggaucuuguucgacagcaU	5	1	JCK-1
......................uggaucuuguucgacagcCc	1	1	LT-1
......................Cggaucuuguucgacagcac	2	1	LT-2
......................uggaucuuguucgacagcUc	2	1	JCK-3
......................uggaucuuguucAacagcac	1	1	LT-2
......................uggaucuuguucgacagUac	1	1	JCK-3
......................uggaucuuguucgacagcac	83	0	JT-3
......................uggaucuuguucgaAagcac	2	1	LCK-2
......................uggaucCuguucgacagcacu	2	1	LT-2
......................uggaucuuguucgaAagcacu	9	1	JCK-3
......................uggaucuuNuucgacagcacu	2	1	JCK-3
......................uggaucuuguucgacagcacA	50	1	LT-2
......................uggaucuugCucgacagcacu	6	1	LCK-3
......................uggaucuuUuucgacagcacu	3	1	JCK-2
......................uggaucuuguucgacagcGcu	1	1	JCK-1
......................uggaucuuguucgacCgcacu	7	1	JCK-1
......................uggCucuuguucgacagcacu	75	1	JCK-3
......................uggaucuuguucgGcagcacu	2	1	LCK-1
......................uggaucuuguucgacagcacC	5	1	JCK-1
......................uggaucuuguucgacagcCcu	1	1	LCK-1
......................uggaucuuAuucgacagcacu	1	1	LT-3
......................uggaucuuguucgacagcacu	2933	0	LT-1
......................uggaucuuguucgacGgcacu	10	1	JCK-3
......................uggaucuuguuGgacagcacu	10	1	JCK-1
......................uggaucuuguucgacagcGcu	1	1	LT-3
......................uggaucuuguucgacaUcacu	2	1	JCK-1

```
                      Mature                                                       Star
cgcauguucuucguuuaccuucuggaucuuguucgacagcacucccaacaacguuguuguuuuggaugauauagaugugcugucgaacaugauccagaaggugcugucgaacaacauacaaaaggggucuacuguacguccggcccaucaugaucuucuucuucuucuucuucuucuuccugaaagaguaucaaacauggggguuucuguuuguuucuucuucuuuuucuuuuuacaaguagauuua
......................uggaucuuguucgacagcaUu............................................................................................................................................................................................................   17      1     LT-3
......................uggaucuuguucgacagcacA............................................................................................................................................................................................................   61      1     LCK-3
......................uggaucuuguucgacagcaUu............................................................................................................................................................................................................   13      1     JCK-1
......................uggaucuuguucgaUagcacu............................................................................................................................................................................................................   2       1     LT-3
......................uUgaucuuguucgacagcacu............................................................................................................................................................................................................   2       1     LCK-3
......................uAgaucuuguucgacagcacu............................................................................................................................................................................................................   3       1     LT-2
......................ugAaucuuguucgacagcacu............................................................................................................................................................................................................   1       1     LT-3
......................uggaucuuguucgacagcacu............................................................................................................................................................................................................   1264    0     LCK-1
......................uggaucuuguucgacagcacC............................................................................................................................................................................................................   22      1     JCK-2
......................uAgaucuuguucgacagcacu............................................................................................................................................................................................................   7       1     JCK-3
......................uggaucuuguucgacCgcacu............................................................................................................................................................................................................   10      1     JCK-3
......................uggaucuugCucgacagcacu............................................................................................................................................................................................................   3       1     JCK-1
......................uggaucuuguucgacagcacA............................................................................................................................................................................................................   59      1     JCK-3
......................uggaucuuAuucgacagcacu............................................................................................................................................................................................................   3       1     LCK-3
......................uggaucuuguucgacCgcacu............................................................................................................................................................................................................   3       1     LT-3
......................uggaucuuguucgacagcacA............................................................................................................................................................................................................   37      1     JT-1
......................uggaucuuguucgCcagcacu............................................................................................................................................................................................................   2       1     JT-3
......................uggaucuCguucgacagcacu............................................................................................................................................................................................................   7       1     JCK-2
......................uggaucuuguucgGcagcacu............................................................................................................................................................................................................   2       1     JCK-3
......................uggauUuuguucgacagcacu............................................................................................................................................................................................................   4       1     JCK-2
......................uggauUuuguucgacagcacu............................................................................................................................................................................................................   1       1     JCK-1
......................uNgaucuuguucgacagcacu............................................................................................................................................................................................................   29      1     JCK-3
......................uggaucuuguucgacagcacu............................................................................................................................................................................................................   1314    0     LCK-2
......................uggCucuuguucgacagcacu............................................................................................................................................................................................................   10      1     LT-1
......................uggaucuuguucgacagcCcu............................................................................................................................................................................................................   11      1     JCK-1
......................uggaucuuguucAacagcacu............................................................................................................................................................................................................   1       1     LCK-1
......................uggaucuuguucgacagcacC............................................................................................................................................................................................................   4       1     JT-3
......................uggaucuuguucgaGagcacu............................................................................................................................................................................................................   1       1     JCK-3
......................uggCucuuguucgacagcacu............................................................................................................................................................................................................   67      1     JCK-1
......................uggCucuuguucgacagcacu............................................................................................................................................................................................................   55      1     LT-2
......................uggCucuuguucgacagcacu............................................................................................................................................................................................................   1       1     LCK-1
......................uggaucuuguucgacagcacC............................................................................................................................................................................................................   1       1     LT-1
......................uggauUuuguucgacagcacu............................................................................................................................................................................................................   4       1     JCK-3
......................uggaucuuguucgacagcUcu............................................................................................................................................................................................................   2       1     LT-1
......................uggaucuuguucgCcagcacu............................................................................................................................................................................................................   48      1     JCK-3
......................ugNaucuuguucgacagcacu............................................................................................................................................................................................................   2       1     LCK-1
......................uggaucuuguucAacagcacu............................................................................................................................................................................................................   7       1     JCK-2
......................uggaucuuguucgacagcNcu............................................................................................................................................................................................................   10      1     JCK-1
......................Cggaucuuguucgacagcacu............................................................................................................................................................................................................   2       1     LT-2
......................uggCucuuguucgacagcacu............................................................................................................................................................................................................   1       1     JT-2
......................ugAaucuuguucgacagcacu............................................................................................................................................................................................................   2       1     LCK-3
......................uggaucuuguucgacagcaAu............................................................................................................................................................................................................   2       1     JCK-3
......................ugNaucuuguucgacagcacu............................................................................................................................................................................................................   1       1     LT-1
......................uNgaucuuguucgacagcacu............................................................................................................................................................................................................   2       1     LT-3
......................uggaucuuguucgacagNacu............................................................................................................................................................................................................   3       1     JCK-3
......................uggaucuuguucgacagUacu............................................................................................................................................................................................................   16      1     JCK-1
......................uAgaucuuguucgacagcacu............................................................................................................................................................................................................   3       1     LT-3
......................Gggaucuuguucgacagcacu............................................................................................................................................................................................................   1       1     JCK-3
......................uUgaucuuguucgacagcacu............................................................................................................................................................................................................   1       1     JCK-1
......................uggaucuuguuNgacagcacu............................................................................................................................................................................................................   1       1     JCK-2
......................uggaucGuguucgacagcacu............................................................................................................................................................................................................   1       1     JCK-3
......................uggaucuuguCcgacagcacu............................................................................................................................................................................................................   2       1     JCK-2
......................uggCucuuguucgacagcacu............................................................................................................................................................................................................   18      1     LCK-3
......................uggGucuuguucgacagcacu............................................................................................................................................................................................................   4       1     JCK-3
......................uggaucuuguucgCcagcacu............................................................................................................................................................................................................   16      1     LT-2
......................uggaucuuguucgacagcacC............................................................................................................................................................................................................   3       1     LT-3
......................uAgaucuuguucgacagcacu............................................................................................................................................................................................................   1       1     JT-1
......................uggaucuuguucgCcagcacu............................................................................................................................................................................................................   80      1     JCK-2
......................uggaucAuguucgacagcacu............................................................................................................................................................................................................   1       1     JCK-2
......................uggaucuuguucgCcagcacu............................................................................................................................................................................................................   10      1     LT-3
......................uggaCcuuguucgacagcacu............................................................................................................................................................................................................   1       1     LCK-3
......................uggaucuuguucCacagcacu............................................................................................................................................................................................................   1       1     JCK-2
......................uggaucuuguucgacagAacu............................................................................................................................................................................................................   1       1     JCK-2
......................uggCucuuguucgacagcacu............................................................................................................................................................................................................   19      1     LT-3
......................uggaucuuguucgacCgcacu............................................................................................................................................................................................................   13      1     JCK-2
......................uggaucuuguucgacagcacC............................................................................................................................................................................................................   2       1     JT-2
......................uggaucuuguucgCcagcacu............................................................................................................................................................................................................   2       1     JT-1
......................Aggaucuuguucgacagcacu............................................................................................................................................................................................................   4       1     LT-2
......................uggaucuuguucgaUagcacu............................................................................................................................................................................................................   12      1     JCK-1
......................Cggaucuuguucgacagcacu............................................................................................................................................................................................................   2       1     JCK-1
```

Mature **Star**

cgcauguucuucguuuaccuucuggaucuuguucgacagcacucccaacaacguuguuguuuuggaugauauagaugugcugucgaacaugauccagaaggugcugucgaacaacauacaaaaaggggucuacuguacguccggcccaucaugaucuucuucuucuucuucuucuucuucuuccugaaagaguaucaaacaugggguuucuguuuguuucuucuucuuuucuuuuuacaaguagauuua

uggaCcuuguucgacagcacu	1	1	JCK-1
uggaucuuguucgacagcaAu	12	1	JCK-1
uggaucuugCucgacagcacu	9	1	JCK-2
uUgaucuuguucgacagcacu	1	1	LT-2
uggaucuuguucgacaUcacu	5	1	JCK-2
uAgaucuuguucgacagcacu	2	1	LCK-2
uggaucuuguuNgacagcacu	121	1	JCK-1
uggaucuuUuucgacagcacu	3	1	JCK-3
uCgaucuuguucgacagcacu	3	1	JCK-2
uggaucuuAuucgacagcacu	10	1	JCK-2
uggaucCuguucgacagcacu	2	1	LCK-3
ugAaucuuguucgacagcacu	11	1	JCK-3
uggaucuGguucgacagcacu	6	1	JCK-2
ugAaucuuguucgacagcacu	2	1	LT-2
uggaucuuguucNacagcacu	1	1	LCK-1
uggaucuuguucgacagcaUu	38	1	JCK-2
uggaucuuguucAacagcacu	6	1	JCK-1
uggaucuuguucgGcagcacu	2	1	JCK-1
uggaucuugCucgacagcacu	3	1	JCK-3
uggaCcuuguucgacagcacu	5	1	JCK-2
uggaucCuguucgacagcacu	5	1	JCK-3
uggaucuuguucCacagcacu	4	1	LCK-3
uggaucuuguucgacagcacG	12	1	JCK-3
uggaucCuguucgacagcacu	2	1	JCK-1
uggaucuuguucgacagcaAu	2	1	LT-3
uggaucuuguucgacagcacA	24	1	LT-3
uAgaucuuguucgacagcacu	1	1	LT-1
uggaucuuguucgacagcCcu	2	1	LT-3
uggaucuuguucgacagGacu	7	1	JCK-1
uggaucuuguucgacagcacu	5544	0	LCK-3
uggaucuuAuucgacagcacu	1	1	JCK-3
uggaucuuguucgacGgcacu	1	1	LT-2
uggaucuuguucgacagcaAu	3	1	LT-2
ugCaucuuguucgacagcacu	1	1	JCK-1
uggaucuuguucgacagcaUu	18	1	LT-1
uggaucuuguucgacagUacu	19	1	JCK-3
uggaucuuguucgacGgcacu	1	1	LCK-3
uggNucuuguucgacagcacu	4	1	JCK-3
uggaucuuguucgacagcacu	1125	0	JT-1
uggaucuuguucgacagcCcu	1	1	JT-1
ugUaucuuguucgacagcacu	1	1	LT-1
uggaucuuguucgacagcacC	8	1	LT-2
uggaucuuguucgacagcCcu	24	1	JCK-2
uggaucuuguucgacagcacu	277	0	JT-2
uggaucuuguucgacagcCcu	7	1	LT-2
uggaucuuguuGgacagcacu	1	1	JCK-2
uggaucuuguucgacaAcacu	1	1	LT-1
uggaucuuguuGgacagcacu	1	1	LT-3
uggaucuuguCcgacagcacu	1	1	JCK-3
uggaucuuguucgacagcCcu	3	1	LT-1
uggCucuuguucgacagcacu	104	1	JCK-2
Cggaucuuguucgacagcacu	1	1	LCK-3
uggaCcuuguucgacagcacu	4	1	JCK-3
uggauNuuguucgacagcacu	1	1	LCK-1
uNgaucuuguucgacagcacu	31	1	JCK-1
uggaucuuguucgacagcacA	16	1	LT-1
uggaucuuguucgaUagcacu	26	1	JCK-2
uggaucuuguCcgacagcacu	2	1	JCK-1
uggaucuuguucgacGgcacu	2	1	JCK-1
uggaucuuguucgacagcacG	12	1	JCK-1
uggGucuuguucgacagcacu	9	1	JCK-1
uggaucuuguucgacaAcacu	1	1	LCK-3
uggaucuuguucgacGgcacu	1	1	LCK-2
uggaucuuguucgacagcacu	26265	0	JCK-3
Aggaucuuguucgacagcacu	1	1	JCK-2
uggaucuuguucgacagcaAu	3	1	LT-1
uggaucuuguucgNcagcacu	5	1	LCK-1
uggaucuuguucUacagcacu	1	1	JCK-1
uggaucuuguucgacagcacu	423	0	JT-3
uggaucuuguucgacagcaAu	3	1	JCK-2

```
                      Mature                                                  Star
cgcauguucuucguuuaccuucuggaucuuguucgacagcacucccaacaacguuguuguuuuggaugauauagaugugcugucgaacaugauccagaaggugcugucgaacaacauacaaaaaggggucuacuguacguccggcccaucaugaucuucuucuucuucuucuucuucuucuuccugaaagaguaucaaacaugggguuucuguuuguuucuucuucuuuucuuuuuacaaguagauuua
......................ugUaucuuguucgacagcacu..............................................................................................................................................................................................  2      1  JCK-1
......................uggauNuuguucgacagcacu..............................................................................................................................................................................................  1      1  JCK-1
......................uggaucuuguucgacagUacu..............................................................................................................................................................................................  1      1  LT-1
......................uggaucuuguCcgacagcacu..............................................................................................................................................................................................  1      1  LT-3
......................uggaucuuCuucgacagcacu..............................................................................................................................................................................................  1      1  JCK-3
......................uUgaucuuguucgacagcacu..............................................................................................................................................................................................  3      1  JCK-2
......................Cggaucuuguucgacagcacu..............................................................................................................................................................................................  2      1  LT-3
......................uggaucuuguucgaUagcacu..............................................................................................................................................................................................  11     1  JCK-3
......................uggNucuuguucgacagcacu..............................................................................................................................................................................................  1      1  JCK-2
......................uggaucuuguucgacagcaNu..............................................................................................................................................................................................  23     1  JCK-1
......................uggaucCuguucgacagcacu..............................................................................................................................................................................................  1      1  JT-3
......................uggaucuuguucgacagcacG..............................................................................................................................................................................................  2      1  LT-3
......................uggaucuuguucgaNagcacu..............................................................................................................................................................................................  4      1  LCK-1
......................ugUaucuuguucgacagcacu..............................................................................................................................................................................................  1      1  LCK-3
......................uAgaucuuguucgacagcacu..............................................................................................................................................................................................  10     1  JCK-2
......................uggaucuuguucgacagUacu..............................................................................................................................................................................................  23     1  JCK-2
......................uggaucuuguucgCcagcacu..............................................................................................................................................................................................  47     1  JCK-1
......................uggaucuuguuGgacagcacu..............................................................................................................................................................................................  1      1  JCK-3
......................ugUaucuuguucgacagcacu..............................................................................................................................................................................................  7      1  JCK-3
......................uggaucuuguucgacagcaGu..............................................................................................................................................................................................  1      1  JCK-1
......................uggCucuuguucgacagcacu..............................................................................................................................................................................................  4      1  JT-1
......................uggaucuuguucgacagcacu..............................................................................................................................................................................................  20810  0  JCK-1
......................uggaucuuguucgacagcGcu..............................................................................................................................................................................................  2      1  JCK-3
......................uggaucCuguucgacagcacu..............................................................................................................................................................................................  1      1  JT-1
......................uggaucuuguucgacagcacC..............................................................................................................................................................................................  10     1  JCK-3
......................uggaucuuguucgacagcCcu..............................................................................................................................................................................................  2      1  LCK-3
......................uggaucuuguucgacagcacA..............................................................................................................................................................................................  51     1  JCK-1
......................uggaucuuguucgCcagcacu..............................................................................................................................................................................................  6      1  LCK-3
......................uCgaucuuguucgacagcacu..............................................................................................................................................................................................  1      1  LT-2
......................uggaucuCguucgacagcacu..............................................................................................................................................................................................  1      1  JCK-1
......................uggaucuuguucAacagcacu..............................................................................................................................................................................................  1      1  LCK-2
......................uggaucuuguucgacagcGcu..............................................................................................................................................................................................  7      1  JCK-2
......................uggaucuuguucgacagcacu..............................................................................................................................................................................................  4453   0  LT-3
......................uggaucuuguucgaAagcacu..............................................................................................................................................................................................  1      1  JCK-1
......................uggaucuCguucgacagcacu..............................................................................................................................................................................................  2      1  JCK-3
......................uggaucuuguucgacagcCcu..............................................................................................................................................................................................  1      1  LCK-2
......................uggaucuuguucgCcagcacu..............................................................................................................................................................................................  8      1  LT-1
......................ugUaucuuguucgacagcacu..............................................................................................................................................................................................  3      1  JCK-2
......................uggaucuuguucgacagcacu..............................................................................................................................................................................................  4196   0  LT-2
......................uggaucuuguucgacagcUcu..............................................................................................................................................................................................  1      1  JCK-3
......................uggNucuuguucgacagcacu..............................................................................................................................................................................................  1      1  LCK-1
......................uggaucuuguucgacagcacG..............................................................................................................................................................................................  15     1  JCK-2
......................uggaucuuguucgacaUcacu..............................................................................................................................................................................................  4      1  JCK-3
......................uggaucuuguucgacagcaUu..............................................................................................................................................................................................  7      1  JT-1
......................Cggaucuuguucgacagcacu..............................................................................................................................................................................................  8      1  JCK-2
......................uggGucuuguucgacagcacu..............................................................................................................................................................................................  3      1  JCK-2
......................uggaucuuguuAgacagcacu..............................................................................................................................................................................................  1      1  JCK-2
......................uggaucuuguucgacCgcacu..............................................................................................................................................................................................  1      1  LCK-2
......................uUgaucuuguucgacagcacu..............................................................................................................................................................................................  6      1  JCK-3
......................uggaucuuguucgacagcacC..............................................................................................................................................................................................  2      1  LCK-3
......................uggaucuuguuAgacagcacu..............................................................................................................................................................................................  1      1  JCK-1
......................uggaucCuguucgacagcacu..............................................................................................................................................................................................  7      1  JCK-2
......................uAgaucuuguucgacagcacu..............................................................................................................................................................................................  9      1  JCK-1
......................uggaucuuguucUacagcacu..............................................................................................................................................................................................  6      1  JCK-3
......................uggaucuuguucgacagcacG..............................................................................................................................................................................................  2      1  LT-1
......................uggaucuuguucgCcagcacu..............................................................................................................................................................................................  2      1  LCK-2
......................uggNucuuguucgacagcacu..............................................................................................................................................................................................  21     1  JCK-1
......................uggGucuuguucgacagcacu..............................................................................................................................................................................................  1      1  LCK-3
......................uggaucuuguucgacagcCcu..............................................................................................................................................................................................  17     1  JCK-3
......................uggCucuuguucgacagcacu..............................................................................................................................................................................................  2      1  LCK-2
......................ugNaucuuguucgacagcacu..............................................................................................................................................................................................  9      1  LT-2
......................uggaucuuguucAacagcacu..............................................................................................................................................................................................  1      1  JCK-3
......................uggaucuuguucgacagcUcu..............................................................................................................................................................................................  1      1  JCK-2
......................uggaucuuguucgacagcacu..............................................................................................................................................................................................  35956  0  JCK-2
......................uggaucuuguucgaUagcacu..............................................................................................................................................................................................  2      1  LT-1
......................uggaucuuguucgacagcacN..............................................................................................................................................................................................  6      1  JCK-1
......................uggaucuuguucgacagcacG..............................................................................................................................................................................................  6      1  LCK-3
......................uNgaucuuguucgacagcacu..............................................................................................................................................................................................  37     1  JCK-2
......................uggaucuuAuucgacagcacu..............................................................................................................................................................................................  1      1  LT-2
......................uggaCcuuguucgacagcacu..............................................................................................................................................................................................  1      1  LCK-2
```

Mature Star

cgcauguucuucguuuaccuucuggaucuuguucgacagcacucccaacaacguuguuguuuuggaugauauagaugugcugucgaacaugauccagaaggugcugucgaacaacauacaaaaggggucuacuguacguccggcccaucaugaucuucuucuucuucuucuucuucuuccugaaagaguaucaaacaugggguuucuguuuguuucuucuucuuuuucuuuuuacaaguagauuua

......................uggaucuuguucgacCgcacu	3	1	LT-2
......................uggaucuuguucgacagcacA	79	1	JCK-2
......................uggaucuuguucgacGgcacu	1	1	LT-1
......................uggaucuuguucgacaUcacu	1	1	LT-2
......................uggaucuuguucgacGgcacu	5	1	JCK-2
......................uggaucuuguucUacagcacu	4	1	JCK-2
......................uggaucuuguucgacagcaUu	17	1	JCK-3
......................uggaucuuguucgacaCcacu	1	1	JCK-1
......................uggaucuCguucgacagcacu	1	1	LT-1
......................uggaucuuguucgacagcaAu	1	1	LCK-2
......................ugAaucuuguucgacagcacu	8	1	JCK-2
......................uggaucuuguucgacagcaUu	17	1	LT-2
......................uggaucuuAuucgacagcacu	2	1	JCK-1
......................uggaucuuguucgacagcaUu	38	1	LCK-3
......................uggaucuuguGcgacagcacu	3	1	JCK-1
......................uAgaucuuguucgacagcacu	7	1	LCK-3
......................uggaucuuguucgacaAcacu	9	1	JCK-2
......................uggaucuuguuNgacagcacu	1	1	JT-1
......................uggaucuuguucgacaAcacu	2	1	JCK-3
......................ugAaucuuguucgacagcacu	2	1	LT-1
......................uggaucuuguucgaAagcacu	5	1	JCK-2
......................ugAaucuuguucgacagcacu	6	1	JCK-1
......................uggaucuuguucgacaAcacu	8	1	JCK-1
......................Cggaucuuguucgacagcacu	2	1	JCK-3
......................uggaucuuguucgacagcaAu	1	1	LCK-3
......................uggaucuuguuAgacagcacu	1	1	LCK-3
......................uggaucuCguucgacagcacu	1	1	LCK-2
......................uCgaucuuguucgacagcacu	1	1	JCK-3
......................uggaucuuguucgacagNacuc	1	1	JCK-3
......................uggaucuuguucgacagcacuc	58	0	JT-2
......................uggaucuuUuucgacagcacuc	3	1	JCK-3
......................ugAaucuuguucgacagcacuc	5	1	JCK-3
......................Cggaucuuguucgacagcacuc	2	1	JCK-1
......................uggaucuuguucgacagUacuc	3	1	JCK-3
......................uggaucCuguucgacagcacuc	2	1	LCK-3
......................uggaucuuguucgCcagcacuc	17	1	JCK-1
......................uggaucuuguucAacagcacuc	2	1	JCK-3
......................uggaucuuguucgacagcacuU	31	1	LCK-3
......................uggaucuuguucgacagcacuG	3	1	LT-1
......................uggaucuuguucgacagcacuU	63	1	JCK-1
......................uggaucuuguucgCcagcacuc	5	1	LT-3
......................uggaucuuguucCacagcacuc	2	1	JCK-1
......................uggCucuuguucgacagcacuc	8	1	LT-2
......................uggaucuuguucgacagcacuU	27	1	LT-1
......................uAgaucuuguucgacagcacuc	4	1	JCK-2
......................uggaucuuguucgacCgcacuc	1	1	LCK-2
......................uggaucuuAuucgacagcacuc	1	1	JCK-3
......................uggaucuuguucgacagcacNc	4	1	JCK-1
......................uggaucuuguucgacagcacAc	1	1	LT-1
......................uggaucuuUuucgacagcacuc	1	1	JCK-1
......................uggaucuuguucgacagGacuc	2	1	JCK-2
......................uNgaucuuguucgacagcacuc	11	1	JCK-1
......................uggaucuuguucgacagUacuc	5	1	JCK-2
......................uggaucuuguucgacaAcacuc	1	1	LT-3
......................uggaucuuguucgacCgcacuc	1	1	LT-3
......................uggaucuuguucgaUagcacuc	10	1	JCK-1
......................uggaucuuguCcgacagcacuc	1	1	JCK-3
......................uUgaucuuguucgacagcacuc	1	1	LT-1
......................ugAaucuuguucgacagcacuc	5	1	LT-3
......................uggaucuuguucgacagcacuU	81	1	JCK-3
......................uggaucuuAuucgacagcacuc	2	1	JCK-2
......................uggaucuuguucgacagcCcuc	1	1	LT-3
......................uggaucuuguucgUcagcacuc	1	1	JCK-1
......................Cggaucuuguucgacagcacuc	1	1	LT-2
......................uAgaucuuguucgacagcacuc	2	1	LT-1
......................uggaucuuguucgacagcacuU	25	1	LT-2
......................uggaucuuguucgacagcaUuc	7	1	JCK-1
......................ugAaucuuguucgacagcacuc	4	1	JCK-2
......................uggaucuuguucUacagcacuc	1	1	JCK-2
......................uggaucuuguucgacagcacuA	67	1	JCK-3

Mature **Star**

cgcauguucuucguuuaccuucuggaucuuguucgacagcacucccaacaacguuguuguuuuggaugauauagaugugcugucgaacaugauccagaaggugcugucgaacaacauacaaaaggggucuacuguacguccggcccaucaugaucuucuucuucuucuucuucuucuucuuccugaaagaguaucaaacaugggguuucuguuuguuucuucuucuuuucuuuuacaaguagauuua

......................uggaucuuguucgaAagcacuc	5	1	JCK-2
......................uggaucuuguucAacagcacuc	1	1	JCK-1
......................uUgaucuuguucgacagcacuc	2	1	JCK-3
......................uggaucuuguucgacagcacuc	8479	0	JCK-1
......................uggaucuuguucgacagcacuN	4	1	JCK-1
......................uggauUuuguucgacagcacuc	3	1	JCK-1
......................uggaucuuguucgacagcacuG	1	1	LT-3
......................uggCucuuguucgacagcacuc	7	1	LT-3
......................uggaucuuguucgGcagcacuc	1	1	LCK-1
......................uggaucuuguucgacagcacuA	9	1	JT-1
......................uggaucuuguucgaUagcacuc	42	1	JCK-2
......................uggaucuuguuNgacagcacuc	29	1	JCK-1
......................uggaucuuguucgacaCcacuc	1	1	LT-1
......................uggaucCuguucgacagcacuc	1	1	JCK-2
......................uggaucuuguucgacagcacuc	188	0	JT-1
......................uggauUuuguucgacagcacuc	2	1	JCK-3
......................uggaucuuguucgacagcacuc	69	0	JT-3
......................uggCucuuguucgacagcacuc	19	1	JCK-1
......................uggNucuuguucgacagcacuc	1	1	JCK-3
......................uNgaucuuguucgacagcacuc	9	1	JCK-3
......................uggaucuuguucUacagcacuc	2	1	JCK-3
......................uggaucuuguucgacagAacuc	1	1	JCK-1
......................uggaucuuguucgacagUacuc	4	1	JCK-1
......................uggaucuuguuGgacagcacuc	3	1	JCK-1
......................uggaucuuguucgacagcacuG	12	1	JCK-2
......................uggaucuuguucgacagcNcuc	3	1	JCK-1
......................uggaucuCguucgacagcacuc	1	1	LCK-3
......................uggaucuuguucgCcagcacuc	29	1	JCK-2
......................uAgaucuuguucgacagcacuc	1	1	LT-3
......................uggaucuuguucgacagcCcuc	2	1	LT-1
......................uggaucuuguucgaUagcacuc	2	1	LT-1
......................uggaucuugCucgacagcacuc	5	1	JCK-1
......................uggaucuuguucAacagcacuc	1	1	LT-2
......................uggaucuuguucgacagcacuA	19	1	LT-1
......................uggaucuuguucgacagcacuc	10812	0	JCK-3
......................uggaucuuguucAacagcacuc	1	1	JCK-2
......................uggaucCuguucgacagcacuc	3	1	JCK-3
......................uggaucuuguucgaAagcacuc	3	1	JCK-3
......................uggaucuuguucgacagcCcuc	3	1	JCK-1
......................uggaucuuguucgacagcCcuc	1	1	LT-2
......................uggaucuuguucgacGgcacuc	1	1	LT-1
......................uggaucuuguucgacaAcacuc	4	1	JCK-2
......................uAgaucuuguucgacagcacuc	3	1	JCK-1
......................uggaucuuguucgacagcacuU	35	1	LT-3
......................uggauUuuguucgacagcacuc	4	1	JCK-2
......................uggaucuuguucgacagcacuc	2023	0	LT-1
......................uggaucuCguucgacagcacuc	1	1	LT-1
......................uggaucuuguucgacagcacGc	1	1	JCK-1
......................uggaCcuuguucgacagcacuc	3	1	JCK-3
......................uggaucuuguucgacagcacCc	2	1	JCK-3
......................uggaucuuguucgacGgcacuc	2	1	JCK-2
......................uggaucuuguucgacagcacuc	74	0	LCK-1
......................uggaucuugCucgacagcacuc	4	1	JCK-3
......................uUgaucuuguucgacagcacuc	1	1	JCK-2
......................uggaucuuguucgacCgcacuc	8	1	JCK-2
......................Cggaucuuguucgacagcacuc	3	1	JCK-3
......................uggaucuuAuucgacagcacuc	1	1	LT-3
......................uggaucuuguucgacagcaUuc	14	1	JCK-2
......................uggaucuuguucgacagcacuA	36	1	LCK-3
......................uggaucuuguucgacagcaUuc	10	1	JCK-3
......................uggaucuuguucAacagcacuc	1	1	LT-1
......................uggaucuuguucgacGgcacuc	2	1	LT-2
......................uggaucuuguucUacagcacuc	2	1	JCK-1
......................uggaucuuguucgUcagcacuc	1	1	JCK-3
......................uggaucuuguucgacagcacuA	11	1	LCK-2
......................uggaucuuguucgacCgcacuc	5	1	JCK-3
......................uggaucuuguucgacaUcacuc	4	1	JCK-2
......................uggaucuuUuucgacagcacuc	3	1	JCK-2
......................uggaucuuguucgacagcacCc	2	1	JCK-2
......................uggaCcuuguucgacagcacuc	1	1	LT-1

```
                      Mature                                                   Star
cgcauguucuucguuuaccuucuggaucuuguucgacagcacucccaacaacguuguuguuuuggaugauauagaugugcugucgaacaugauccagaaggugcugucgaacaacauacaaaaaggggucuacuguacguccggcccaucaugaucuucuucuucuucuucuucuucuuccugaaagaguaucaaacaugggguuucuguuuguuucuucuucuuuucuuuuacaaguagauuua
......................uNgaucuuguucgacagcacuc.........................................................................................................................................................................................................   1       1   LT-3
......................uggaucuuguucgacagcCcuc.........................................................................................................................................................................................................   9       1   JCK-2
......................uggaucuuguucgacagcacuc.........................................................................................................................................................................................................   84      0   LCK-2
......................ugNaucuuguucgacagcacuc.........................................................................................................................................................................................................   2       1   LT-2
......................uggCucuuguucgacagcacuc.........................................................................................................................................................................................................   3       1   LT-1
......................uggaucuuguucUacagcacuc.........................................................................................................................................................................................................   2       1   LT-1
......................uggaucuuguucgacagcacCc.........................................................................................................................................................................................................   1       1   LT-3
......................uggGucuuguucgacagcacuc.........................................................................................................................................................................................................   4       1   JCK-1
......................uggaucuuNuucgacagcacuc.........................................................................................................................................................................................................   1       1   JCK-3
......................uggaucuuguucgCcagcacuc.........................................................................................................................................................................................................   1       1   LT-2
......................uggaucuuguucgacagcacuc.........................................................................................................................................................................................................   955     0   LCK-3
......................uggaucuuguucgacagcacuA.........................................................................................................................................................................................................   47      1   JCK-1
......................uggaucuuguGcgacagcacuc.........................................................................................................................................................................................................   5       1   JCK-1
......................uggaucuuAuucgacagcacuc.........................................................................................................................................................................................................   1       1   JCK-1
......................uggaucuuguucgaUagcacuc.........................................................................................................................................................................................................   26      1   JCK-3
......................uNgaucuuguucgacagcacuc.........................................................................................................................................................................................................   15      1   JCK-2
......................uggGucuuguucgacagcacuc.........................................................................................................................................................................................................   2       1   JCK-2
......................uAgaucuuguucgacagcacuc.........................................................................................................................................................................................................   1       1   LCK-3
......................uggaucuuguucgacCgcacuc.........................................................................................................................................................................................................   3       1   JCK-1
......................uggaucuuguucgacaAcacuc.........................................................................................................................................................................................................   1       1   LT-1
......................uUgaucuuguucgacagcacuc.........................................................................................................................................................................................................   1       1   LCK-3
......................uggaucuuguucgacagcacuc.........................................................................................................................................................................................................   2355    0   LT-3
......................ugAaucuuguucgacagcacuc.........................................................................................................................................................................................................   1       1   LT-1
......................uggaCcuuguucgacagcacuc.........................................................................................................................................................................................................   2       1   JCK-2
......................uggaucuuguucgacGgcacuc.........................................................................................................................................................................................................   1       1   JCK-1
......................uAgaucuuguucgacagcacuc.........................................................................................................................................................................................................   2       1   LT-2
......................uggNucuuguucgacagcacuc.........................................................................................................................................................................................................   8       1   JCK-1
......................uggaucCuguucgacagcacuc.........................................................................................................................................................................................................   1       1   LT-3
......................uggaucuuguucgacagcacuc.........................................................................................................................................................................................................   1033    0   LT-2
......................uggaucuuguucgacagcacuA.........................................................................................................................................................................................................   61      1   JCK-2
......................uggaucuuguucgacagcacuU.........................................................................................................................................................................................................   120     1   JCK-2
......................uggaCcuuguucgacagcacuc.........................................................................................................................................................................................................   1       1   JCK-1
......................uggaucuuguucgacagcacuA.........................................................................................................................................................................................................   12      1   LT-2
......................uggaucuuguucgacagcacAc.........................................................................................................................................................................................................   1       1   JCK-2
......................uggaucuuguucgacagcCcuc.........................................................................................................................................................................................................   1       1   JCK-3
......................uggGucuuguucgacagcacuc.........................................................................................................................................................................................................   1       1   LT-3
......................uggCucuuguucgacagcacuc.........................................................................................................................................................................................................   1       1   LCK-3
......................uggaucuuguucgacagcacuG.........................................................................................................................................................................................................   2       1   JCK-3
......................uggCucuuguucgacagcacuc.........................................................................................................................................................................................................   2       1   JT-1
......................uggaucuuguucgUcagcacuc.........................................................................................................................................................................................................   3       1   JCK-2
......................uggaucuuguucgacGgcacuc.........................................................................................................................................................................................................   4       1   JCK-3
......................uggaucuugCucgacagcacuc.........................................................................................................................................................................................................   2       1   LT-1
......................uNgaucuuguucgacagcacuc.........................................................................................................................................................................................................   1       1   LCK-3
......................uAgaucuuguucgacagcacuc.........................................................................................................................................................................................................   3       1   JCK-3
......................uggaucuuguucgacagcacuA.........................................................................................................................................................................................................   14      1   LCK-1
......................uggaucuuguucgaUagcacuc.........................................................................................................................................................................................................   1       1   LT-3
......................uUgaucuuguucgacagcacuc.........................................................................................................................................................................................................   1       1   JCK-1
......................uggaucuCguucgacagcacuc.........................................................................................................................................................................................................   1       1   JCK-3
......................ugAaucuuguucgacagcacuc.........................................................................................................................................................................................................   2       1   JCK-1
......................uggaucuuguucgacagGacuc.........................................................................................................................................................................................................   6       1   JCK-1
......................uggaucuuguucgacagcacuc.........................................................................................................................................................................................................   14605   0   JCK-2
......................uggaucuuguucCacagcacuc.........................................................................................................................................................................................................   1       1   JCK-3
......................uggCucuuguucgacagcacuc.........................................................................................................................................................................................................   43      1   JCK-2
......................uggaucuuguucgCcagcacuc.........................................................................................................................................................................................................   11      1   JCK-3
......................uggaucuuguucgacaUcacuc.........................................................................................................................................................................................................   1       1   JCK-1
......................uggaucuugCucgacagcacuc.........................................................................................................................................................................................................   5       1   JCK-2
......................uggCucuuguucgacagcacuc.........................................................................................................................................................................................................   24      1   JCK-3
......................uggaucuuguucgacagcacuA.........................................................................................................................................................................................................   24      1   LT-3
......................uggaucuuguucCacagcacuc.........................................................................................................................................................................................................   5       1   JCK-2
......................uggaucuuguucgacagcaUuc.........................................................................................................................................................................................................   1       1   LT-1
......................uggaucuuguucgacagcGcuc.........................................................................................................................................................................................................   1       1   JCK-3
......................uggaucuuguucgacaUcacuc.........................................................................................................................................................................................................   1       1   JCK-3
......................uggaucuuguucgacagcaAuc.........................................................................................................................................................................................................   1       1   JCK-1
......................uggaucuuguucgacagcGcuc.........................................................................................................................................................................................................   2       1   JCK-1
......................uggaucuuguucgacagcacuG.........................................................................................................................................................................................................   9       1   JCK-1
......................uggaucuuguucgCcagcacuc.........................................................................................................................................................................................................   2       1   LT-1
......................uggaucuCguucgacagcacuc.........................................................................................................................................................................................................   1       1   JCK-1
......................uggaucuuguucgacagcacuU.........................................................................................................................................................................................................   1       1   LCK-1
......................Cggaucuuguucgacagcacuc.........................................................................................................................................................................................................   3       1   JCK-2
......................uggaucuuguucgacagcaNuc.........................................................................................................................................................................................................   5       1   JCK-1
```

Mature Star

cgcauguucuucguuuaccuucuggaucuuguucgacagcacucccaacaacguuguuguuuuggaugauauagaugugcugucgaacaugauccagaaggugcugucgaacaacauacaaaaggggucuacuguacguccggcccaucaugaucuucuucuucuucuucuucuucuucuuccugaaagaguaucaaacaugggguuucuguuuguuucuucuucuuuucuuuuuacaaguagauuua			
......................uggaucuuguucgacaAcacuc	1	1	JCK-1
......................uggaucuuguucgacagcacucA	59	1	JCK-1
......................uggaucuuguucgacagcacucA	8	1	LT-2
......................uggaucuuguucgacagcacuGc	1	1	JCK-1
......................uggaucuuguucgacagcacuGc	1	1	JCK-2
......................uggaucuuguucgacagcacucG	4	1	JCK-2
......................uggaucuuguucgacagcacuUc	23	1	LCK-1
......................uggaucuuguucgacagcacucG	1	1	JCK-3
......................uggaucuuguucgacagcacucA	18	1	LCK-3
......................uggaucuuguucgacagcacucc	3	0	JCK-3
......................uggaucuuguucgacagGacucc	1	1	JCK-1
......................uggaucuuguucgacagcacucG	5	1	LT-2
......................uggaucuuguucgacagcacucc	3	0	JCK-1
......................uggaucuuguucgacagcacucG	1	1	LT-3
......................uggaucuuguucgacagcacucc	5	0	LCK-3
......................uggaucuuguucgacagcacucc	4	0	JCK-2
......................uggaucuuguucgacagcacucA	64	1	JCK-2
......................uggaucuuguucgacagcacucA	14	1	LT-1
......................uggaucuuguucgacagcacucc	1	0	LT-1
......................uggaucuuguucgacagcacuUc	28	1	LCK-2
......................uggaucuuguucgacagcacucA	11	1	LT-3
......................uggaucuuguucgacagcacuUc	4	1	JCK-3
......................uggaucuuguucgacagcacuUc	2	1	JCK-1
......................uggaucuuguucgacagcacucA	52	1	JCK-3
......................uggaucuuguucgacagcacucN	1	1	JCK-3
......................uggaucuuguucgacagcacucc	1	0	LT-3
......................uggaucuuguucgacagcacuAc	6	1	LT-2
......................uggaucuuguucgacagcacuccc	3	0	JCK-3
......................uggaucuuguucgacagcacuccc	3	0	JCK-2
......................uggaucuuguucgacagcacuccU	4	1	LT-2
......................uggaucuuguucgacagcacuccU	3	1	LT-1
......................uggaucuuguucgacagcacuccc	3	0	JCK-1
......................uggaucuuguucgacagcacucccU	1	1	LT-1
......................uggaucuuguucgacagcacuccGa	2	1	JCK-2
......................uggaucuuguucgacagcacuccGa	2	1	JCK-1
......................uggaucuuguucgacagcacucUca	1	1	LT-3
.......................ggaucuuguucgacagca	10	0	JT-1
.......................ggaucuuguucgacagca	2	0	JCK-1
.......................ggaucuuguucgacagca	5	0	LT-2
.......................Ugaucuuguucgacagcacu	1	1	LT-1
.......................ggaucuuguucgacagcacu	1	0	LT-1
.......................ggaucuuguucgacagcacu	5	0	JCK-3
.......................ggaucuuguucgacagcacu	1	0	JCK-2
.......................ggaucuuguucgacagcacu	1	0	LT-2
.......................ggaucuuguucgacagcacu	2	0	JCK-1
.......................ggaucuuguucgacagcacuc	2	0	JCK-3
.......................ggaucuuguucgacagcacuc	3	0	JCK-2
.......................ggaucuuguucgacagcacuc	1	0	LT-1
........................gaucuuguucgacagcacu	2	0	JCK-3
........................gaucuuguucgacagcacu	2	0	JCK-1
........................gaucuuguucgacagcacucc	6	0	JCK-3
........................gaucuuguucgacagcacucc	3	0	JCK-1
........................gaucuuguucgacagcacucc	8	0	JCK-2
.........................aucuuguucgacagcacu	1	0	LT-3
.........................aucuuguucgacagcacuc	2	0	JCK-1
.........................aucuuguucgacagcacuc	3	0	JCK-3
.........................aucuuguucgacagcacuc	1	0	LT-3
.........................aucuuguucgacagcacuc	2	0	JCK-2
.........................Cucuuguucgacagcacucc	1	1	JCK-2
.........................aucuuguucgacagcacucc	45	0	JCK-2
.........................aucuuguucgacagcacucc	21	0	JCK-1
.........................aucuuguucgCcagcacucc	1	1	JCK-2
.........................aucuuguucgacagcacucc	36	0	JCK-3
.........................aucuuguucgacagcacucc	6	0	LT-1
.........................aucuuguucgacagcacuccc	109	0	JCK-2
.........................aucuuguucgacagcacuUcc	1	1	JCK-2
.........................aucuuguucgacagcacuccc	98	0	JCK-3
.........................aucuuguucgacagcacuUcc	1	1	JCK-1
.........................aucuCguucgacagcacuccc	1	1	JCK-2
.........................aucuuguucgacagcacuccc	17	0	JT-1

```
              Mature                                   Star
cgcauguucuucguuuaccuucuggaucuuguucgacagcacucccaacaacguuguuguuuuggaugauauagaugugcugucgaacaugauccagaaggugcugucgaacaacauacaaaaaggggucuacuguacguccggcccaucaugaucuucuucuucuucuucuucuucuucuuccugaaagaguaucaaacaugggguuucuguuuguuucuucuucuuuucuuuuuacaaguagauuua
.........................aucuuguucgaAagcacuccc...........................................................................................................................................................................................   1    1    JCK-1
.........................Cucuuguucgacagcacuccc...........................................................................................................................................................................................   1    1    JCK-2
.........................aucuuguucgacagcacuccc...........................................................................................................................................................................................   72   0    JCK-1
.........................aucuuguucgacagcacuccca..........................................................................................................................................................................................   1    0    JCK-2
..........................ucuuguucgacagcacuc.............................................................................................................................................................................................   1    0    LT-1
..........................ucuuguucgacagcacuc.............................................................................................................................................................................................   2    0    LT-3
..........................ucuuguucgacagcacucc............................................................................................................................................................................................   1    0    JCK-1
..........................ucuuguucgacagcacucc............................................................................................................................................................................................   4    0    JCK-3
..........................ucuuguucgacagcacucc............................................................................................................................................................................................   5    0    JCK-2
..........................ucuuguucgacagcacuccc...........................................................................................................................................................................................   10   0    JCK-1
..........................ucuuguucgacagcacuccc...........................................................................................................................................................................................   1    0    LT-3
..........................ucuuguucgacagcacuccc...........................................................................................................................................................................................   10   0    JCK-3
..........................ucuuguucgacagcacuccc...........................................................................................................................................................................................   18   0    JCK-2
..........................ucuuguucgacagcCcuccc...........................................................................................................................................................................................   1    1    JCK-2
..........................ucuuguucgacagcacuccca..........................................................................................................................................................................................   2    0    JCK-2
..........................ucuuguucgacagcacuccca..........................................................................................................................................................................................   1    0    JCK-3
..........................ucuuguucgacagcacucccaac........................................................................................................................................................................................   1    0    JCK-3
............................uuguucgacagcacucccaacG.......................................................................................................................................................................................   1    1    JCK-3
...........................................................uuuuggaugauauagaAgugcugucgaaca................................................................................................................................................................   1    1    JCK-3
...........................................................uuuuggaugauauagaAgugcugucgaaca................................................................................................................................................................   1    1    JCK-2
....................................................................auauagaugugcugucgaaca................................................................................................................................................................   7    0    LCK-3
......................................................................auagaugugcugucgaacaug..............................................................................................................................................................   1    0    LT-1
......................................................................auagaugugcugucgaacaugauccag........................................................................................................................................................   1    0    LT-3
.......................................................................uagaugugcugucgaacaugauA...........................................................................................................................................................   2    1    JCK-2
.......................................................................uagaugugcugucgaacaugauA...........................................................................................................................................................   1    1    JCK-1
........................................................................agaugugcugucgaacaug..............................................................................................................................................................   1    0    LT-3
..........................................................................augugcugucgaacaugU.............................................................................................................................................................   3    1    LT-2
..........................................................................augugcugucgaacauga.............................................................................................................................................................   3    0    LCK-3
..........................................................................augugcugucgaacaugauc...........................................................................................................................................................   2    0    LT-3
..........................................................................augugcugucgaacaugaucA..........................................................................................................................................................   1    1    LT-1
..........................................................................aCgugcugucgaacaugaucc..........................................................................................................................................................   4    1    LT-2
..........................................................................augugcugucgaacaugauccaga.......................................................................................................................................................   3    0    LCK-3
..........................................................................aNgugcugucgaacaugauccaga.......................................................................................................................................................   1    1    JCK-3
..........................................................................aCgugcugucgaacaugauccaga.......................................................................................................................................................   1    1    JCK-3
..........................................................................augugcugucgaacaugauccaga.......................................................................................................................................................   3    0    JCK-1
..........................................................................augugcugucgaacaugauccaga.......................................................................................................................................................   1    0    JCK-2
..........................................................................augugcugucgaacaugauccaga.......................................................................................................................................................   4    0    JCK-3
..........................................................................augugcugucgaacaugauccaga.......................................................................................................................................................   2    0    LT-3
...........................................................................ugugcugucgaacaugauA...........................................................................................................................................................   2    1    LT-1
...........................................................................Cgugcugucgaacaugaucc..........................................................................................................................................................   1    1    LT-1
...........................................................................ugugcugucgaacaugaucca.........................................................................................................................................................   1    0    LT-1
...........................................................................ugugcugucgaacaugaucca.........................................................................................................................................................   5    0    JCK-2
...........................................................................ugugcugucgaacaugaucca.........................................................................................................................................................   5    0    JCK-3
...........................................................................ugugcugucgaacaugaucca.........................................................................................................................................................   4    0    LT-3
...........................................................................ugugcugucgaacaugaucca.........................................................................................................................................................   6    0    JCK-1
...........................................................................ugugcugucgaacaugaucca.........................................................................................................................................................   8    0    LT-2
...........................................................................ugugcugucgaacaugauccaga.......................................................................................................................................................   2    0    LT-2
...........................................................................ugugcugucgaacaugauccaga.......................................................................................................................................................   12   0    LCK-3
...........................................................................uCugcugucgaacaugauccaga.......................................................................................................................................................   1    1    LT-1
............................................................................gugcugucgGacaugauc...........................................................................................................................................................   6    1    JCK-2
............................................................................gugcugucgaacaugauA...........................................................................................................................................................   1    1    LT-3
............................................................................gugcugucgaacaugauA...........................................................................................................................................................   1    1    LT-1
............................................................................gugcugucgGacaugauc...........................................................................................................................................................   3    1    JCK-1
............................................................................gugcugucgGacaugauc...........................................................................................................................................................   5    1    JCK-3
............................................................................gugcugucgaacaugauUc..........................................................................................................................................................   1    1    LT-1
............................................................................gugcugucgaCcaugaucc..........................................................................................................................................................   1    1    LT-2
............................................................................gugcugucgaacaugaucca.........................................................................................................................................................   14   0    JCK-2
............................................................................gugcugucgaacaugaucca.........................................................................................................................................................   4    0    JCK-3
............................................................................gugcugucgaacaugaucca.........................................................................................................................................................   7    0    JCK-1
............................................................................gugcugucgaacaugaucca.........................................................................................................................................................   12   0    LT-1
............................................................................gugcugucgaGcaugaucca.........................................................................................................................................................   1    1    LT-3
............................................................................gugcugucgaacaugaucca.........................................................................................................................................................   18   0    LT-2
............................................................................gugcugucgaacaugaucca.........................................................................................................................................................   9    0    LCK-3
............................................................................gugcugucgaacaugaucca.........................................................................................................................................................   16   0    LT-3
............................................................................gugcugucgGacaugaucca.........................................................................................................................................................   1    1    JCK-2
............................................................................gugcugucgaacaugauccU.........................................................................................................................................................   1    1    LT-2
............................................................................gugcugucgGacaugauccag........................................................................................................................................................   2    1    JCK-1
............................................................................gugcugucgaacaugauccag........................................................................................................................................................   24   0    JCK-3
............................................................................gugcugucgGacaugauccag........................................................................................................................................................   2    1    JCK-3
............................................................................gugcugucgaacaugauccag........................................................................................................................................................   15   0    JCK-2
```

```
               Mature                              Star
cgcauguucuucguuuaccuucuggaucuuguucgacagcacucccaacaacguuguuguuuuggaugauauagaugugcugucgaacaugauccagaaggugcugucgaacaacauacaaaaaggggucuacuguacguccggcccaucaugaucuucuucuucuucuucuucuucuuccugaaagaguaucaaacaugggguuucuguuuguuucuucuucuuuuucuuuuuacaaguagauuua
............................................................................gugcugucgaacaugauccag......................................................................................................................................................  10    0    LT-2
............................................................................gugcugucgaacaugauccag......................................................................................................................................................  17    0    LT-1
............................................................................gugcugucgaacaugauccag......................................................................................................................................................  9     0    JCK-1
............................................................................gugcugucgaacaugauccag......................................................................................................................................................  14    0    LT-3
............................................................................Cugcugucgaacaugauccag......................................................................................................................................................  1     1    LT-3
............................................................................gugcugucgaacaugauccag......................................................................................................................................................  6     0    LCK-3
............................................................................gugcugucgaacaugauccaA......................................................................................................................................................  1     1    JCK-2
............................................................................gugcugucgGacaugauccag......................................................................................................................................................  5     1    JCK-2
............................................................................gugcugucgaacaugauccaga.....................................................................................................................................................  1     0    JCK-1
............................................................................Cugcugucgaacaugauccaga.....................................................................................................................................................  2     1    JCK-1
............................................................................gugcugucgaacaugauccaga.....................................................................................................................................................  2     0    JCK-3
............................................................................Cugcugucgaacaugauccaga.....................................................................................................................................................  1     1    JCK-3
............................................................................gugcugucgaacaugauccagU.....................................................................................................................................................  1     1    LT-3
............................................................................gugcugucgaacaugauccagU.....................................................................................................................................................  1     1    LT-1
............................................................................gugcugucgGacaugauccaga.....................................................................................................................................................  3     1    JCK-1
............................................................................Cugcugucgaacaugauccaga.....................................................................................................................................................  2     1    JCK-2
............................................................................gugcugucgGacaugauccaga.....................................................................................................................................................  1     1    JCK-2
............................................................................Cugcugucgaacaugauccaga.....................................................................................................................................................  2     1    LT-3
............................................................................gugcugucgaacaugauccagU.....................................................................................................................................................  2     1    JCK-1
............................................................................gugcugucgaacaugauccaga.....................................................................................................................................................  1     0    JCK-2
............................................................................gugcugucgaacaugauccagU.....................................................................................................................................................  1     1    LCK-3
.............................................................................ugcugucgaacaugaucA........................................................................................................................................................  4     1    LT-1
.............................................................................ugcugucgaacaugaucU........................................................................................................................................................  31    1    LCK-1
.............................................................................ugcugucgaacaugaucU........................................................................................................................................................  2     1    LT-2
.............................................................................ugcugucgGacaugaucc........................................................................................................................................................  112   1    JCK-1
.............................................................................ugcugucgaacaugaucU........................................................................................................................................................  1     1    JCK-2
.............................................................................ugcugucgaacaugaucU........................................................................................................................................................  19    1    LCK-2
.............................................................................ugcugucgGacaugaucc........................................................................................................................................................  28    1    JT-1
.............................................................................ugcugucgaacCugaucc........................................................................................................................................................  1     1    LCK-3
.............................................................................ugcugucgaacaugaucU........................................................................................................................................................  2     1    LT-3
.............................................................................ugcugucgGacaugaucc........................................................................................................................................................  5     1    JT-2
.............................................................................ugcugucgaacaugaucU........................................................................................................................................................  16    1    LCK-3
.............................................................................ugcugucgGacaugaucc........................................................................................................................................................  195   1    JCK-2
.............................................................................ugcugucgGacaugaucc........................................................................................................................................................  140   1    JCK-3
.............................................................................ugcugucgaacaugaucU........................................................................................................................................................  1     1    LT-1
.............................................................................ugcugucgGacaugaucc........................................................................................................................................................  3     1    JT-3
.............................................................................ugcugucgaacaugaucA........................................................................................................................................................  1     1    LT-3
.............................................................................ugcugucgaacaugaucca.......................................................................................................................................................  6     0    JT-2
.............................................................................uAcugucgaacaugaucca.......................................................................................................................................................  1     1    LCK-3
.............................................................................ugcugucgaacaugaucca.......................................................................................................................................................  26    0    JCK-2
.............................................................................ugcugucgGacaugaucca.......................................................................................................................................................  59    1    JCK-2
.............................................................................ugcugucgaacaugaCcca.......................................................................................................................................................  1     1    LCK-3
.............................................................................ugcugucgaacaugaucca.......................................................................................................................................................  4     0    JT-3
.............................................................................ugcugucgaaAaugaucca.......................................................................................................................................................  1     1    LT-2
.............................................................................ugcugucgaacGugaucca.......................................................................................................................................................  1     1    LCK-3
.............................................................................ugcugucgaacaugaucca.......................................................................................................................................................  53    0    LCK-1
.............................................................................ugcugucgaacaugaucca.......................................................................................................................................................  81    0    LCK-3
.............................................................................ugcugucgaacaugaucca.......................................................................................................................................................  24    0    JCK-1
.............................................................................ugcugucgGacaugaucca.......................................................................................................................................................  41    1    JCK-1
.............................................................................ugcugucgaacaugaucca.......................................................................................................................................................  18    0    JCK-3
.............................................................................ugcugucgGacaugaucca.......................................................................................................................................................  30    1    JCK-3
.............................................................................ugcugucgaacaugaucca.......................................................................................................................................................  61    0    LT-1
.............................................................................ugcugucgaacaugaucca.......................................................................................................................................................  67    0    JT-1
.............................................................................ugcugucgaacaugaucca.......................................................................................................................................................  50    0    LCK-2
.............................................................................ugcugucgaacaugCucca.......................................................................................................................................................  1     1    LT-2
.............................................................................ugcugucgaacaugaucca.......................................................................................................................................................  96    0    LT-3
.............................................................................ugcugucgaacaugauccU.......................................................................................................................................................  1     1    LT-1
.............................................................................ugcugucgGacaugaucca.......................................................................................................................................................  3     1    JT-3
.............................................................................ugcugucgaacaugaucca.......................................................................................................................................................  125   0    LT-2
.............................................................................ugcugucgGacaugaucca.......................................................................................................................................................  7     1    JT-1
.............................................................................ugcugucgaacaugauccag......................................................................................................................................................  114   0    LCK-1
.............................................................................ugcugucgGacaugauccag......................................................................................................................................................  49    1    JT-1
.............................................................................uNcugucgaacaugauccag......................................................................................................................................................  1     1    JCK-3
.............................................................................ugcugucgaCcaugauccag......................................................................................................................................................  1     1    LT-2
.............................................................................ugcugucgaacaugauccag......................................................................................................................................................  45    0    JT-2
.............................................................................ugcugucgaacaugGuccag......................................................................................................................................................  1     1    JT-1
.............................................................................ugcugucgaacaugauccag......................................................................................................................................................  117   0    LCK-2
.............................................................................ugcugucgaacaugauccaU......................................................................................................................................................  1     1    JCK-1
.............................................................................ugcugucgaacaugCuccag......................................................................................................................................................  1     1    LT-2
.............................................................................ugcugucgaacaugauccaA......................................................................................................................................................  2     1    JCK-2
```

Mature Star

```
cgcauguucuucguuuaccuucuggaucuuguucgacagcacucccaacaacguuguuguuuuggaugauauagaugugcugucgaacaugauccagaaggugcugucgaacaacauacaaaaaggggucuacuguacguccggcccaucaugaucuucuucuucuucuucuucuucuucuuccugaaagaguaucaaacaugggguuucuguuuguuucuucuucuuuuucuuuuuacaaguagauuua
.............................................................................ugcugucAaacaugauccag.........................................................................................................................................................   1      1    LT-2
.............................................................................ugcugucgaacCugauccag.........................................................................................................................................................   1      1    LCK-2
.............................................................................ugcugAcgaacaugauccag.........................................................................................................................................................   1      1    LT-3
.............................................................................ugcugucgaacaugCuccag.........................................................................................................................................................   1      1    LT-3
.............................................................................ugcugucgaacCugauccag.........................................................................................................................................................   1      1    LT-2
.............................................................................ugcugCcgaacaugauccag.........................................................................................................................................................   1      1    LT-3
.............................................................................ugcNgucgaacaugauccag.........................................................................................................................................................   1      1    JCK-1
.............................................................................ugcugucgGacaugauccag.........................................................................................................................................................   497    1    JCK-3
.............................................................................ugcugucgaacaugauccag.........................................................................................................................................................   143    0    JCK-1
.............................................................................ugcugucgCacaugauccag.........................................................................................................................................................   2      1    LT-2
.............................................................................ugcugucgaacaugCuccag.........................................................................................................................................................   1      1    LT-1
.............................................................................ugcugucgaacaugauccag.........................................................................................................................................................   73     0    JT-3
.............................................................................ugcugucgGacaugauccag.........................................................................................................................................................   431    1    JCK-1
.............................................................................uNcugucgaacaugauccag.........................................................................................................................................................   1      1    JT-1
.............................................................................ugcugucgaacaugauccag.........................................................................................................................................................   40     0    JT-1
.............................................................................ugcugucgaacaugauccaA.........................................................................................................................................................   2      1    LT-3
.............................................................................ugcugucgaacaugauccCg.........................................................................................................................................................   1      1    LT-3
.............................................................................ugcugucgaacaugauccaA.........................................................................................................................................................   2      1    JCK-3
.............................................................................ugNugucgaacaugauccag.........................................................................................................................................................   1      1    LT-2
.............................................................................uAcugucgaacaugauccag.........................................................................................................................................................   1      1    LT-2
.............................................................................ugcugucgaacaugauccag.........................................................................................................................................................   530    0    LT-3
.............................................................................ugcugucgaacaugauccag.........................................................................................................................................................   262    0    JCK-2
.............................................................................ugcugucgaCcaugauccag.........................................................................................................................................................   1      1    LCK-2
.............................................................................ugcugucgCacaugauccag.........................................................................................................................................................   1      1    JCK-3
.............................................................................ugcugucgaCcaugauccag.........................................................................................................................................................   1      1    LCK-3
.............................................................................ugcugucgaCcaugauccag.........................................................................................................................................................   1      1    LT-3
.............................................................................ugcugucgaacCugauccag.........................................................................................................................................................   2      1    LT-1
.............................................................................ugcugucgGacaugauccag.........................................................................................................................................................   64     1    JT-3
.............................................................................ugcugucgCacaugauccag.........................................................................................................................................................   1      1    LCK-3
.............................................................................ugcugucgGacaugauccag.........................................................................................................................................................   787    1    JCK-2
.............................................................................ugcugucgaacaugauccag.........................................................................................................................................................   458    0    LT-2
.............................................................................ugcugucgaacaugauccag.........................................................................................................................................................   441    0    LT-1
.............................................................................ugcugucgaacaugauccag.........................................................................................................................................................   487    0    LCK-3
.............................................................................ugcugucgCacaugauccag.........................................................................................................................................................   1      1    LT-1
.............................................................................ugcugucgaacaugauccaC.........................................................................................................................................................   1      1    LT-3
.............................................................................ugcugucgaacaugauccag.........................................................................................................................................................   169    0    JCK-3
.............................................................................ugcugucgGacaugauccag.........................................................................................................................................................   29     1    JT-2
.............................................................................ugcugucgaacaugauccaN.........................................................................................................................................................   1      1    JCK-1
.............................................................................ugcugucgaacaCgauccaga........................................................................................................................................................   1      1    LT-1
.............................................................................ugcugucgaacaugauccagU........................................................................................................................................................   2      1    JCK-2
.............................................................................ugcugucgGacaugauccaga........................................................................................................................................................   20     1    JT-3
.............................................................................ugcugucgaacaugGuccaga........................................................................................................................................................   1      1    JCK-3
.............................................................................ugcugucgaacaugauccaga........................................................................................................................................................   2163   0    JCK-3
.............................................................................ugcugucgaacaugauccaga........................................................................................................................................................   125    0    LCK-1
.............................................................................ugcugucgaacaugauccaga........................................................................................................................................................   145    0    JT-3
.............................................................................ugcugucgaacaugauccaga........................................................................................................................................................   186    0    JT-1
.............................................................................ugcugucUaacaugauccaga........................................................................................................................................................   1      1    LT-1
.............................................................................ugcugucAaacaugauccaga........................................................................................................................................................   1      1    JCK-2
.............................................................................ugcugucgaCcaugauccaga........................................................................................................................................................   3      1    JCK-2
.............................................................................ugGugucgaacaugauccaga........................................................................................................................................................   1      1    LT-2
.............................................................................ugcugucgaaAaugauccaga........................................................................................................................................................   1      1    LT-1
.............................................................................ugcugucgaacaugauccaga........................................................................................................................................................   2920   0    JCK-2
.............................................................................ugcugucgaacaugaCccaga........................................................................................................................................................   1      1    LT-3
.............................................................................uNcugucgaacaugauccaga........................................................................................................................................................   3      1    JCK-1
.............................................................................ugcuguAgaacaugauccaga........................................................................................................................................................   1      1    LT-1
.............................................................................ugcugucgGacaugauccaga........................................................................................................................................................   12782  1    JCK-2
.............................................................................ugcuAucgaacaugauccaga........................................................................................................................................................   1      1    LT-3
.............................................................................ugcugucgaacauAauccaga........................................................................................................................................................   2      1    LT-1
.............................................................................ugcugucgaacCugauccaga........................................................................................................................................................   5      1    JCK-2
.............................................................................ugcugucgaacCugauccaga........................................................................................................................................................   2      1    LT-3
.............................................................................uAcugucgaacaugauccaga........................................................................................................................................................   4      1    LT-3
.............................................................................ugcugucgaCcaugauccaga........................................................................................................................................................   1      1    JCK-3
.............................................................................ugcugucgaacaugauccaga........................................................................................................................................................   2567   0    LT-3
.............................................................................ugcugucgaacCugauccaga........................................................................................................................................................   1      1    LT-1
.............................................................................ugcugucgaCcaugauccaga........................................................................................................................................................   1      1    LCK-3
.............................................................................ugcugucgaacaugauccaga........................................................................................................................................................   107    0    LCK-2
.............................................................................Cgcugucgaacaugauccaga........................................................................................................................................................   1      1    LT-1
.............................................................................ugcugucAaacaugauccaga........................................................................................................................................................   1      1    LT-1
.............................................................................ugcugucgaacaugauccaga........................................................................................................................................................   1025   0    LCK-3
.............................................................................ugcugucgaacaugaucUaga........................................................................................................................................................   1      1    LT-3
```

```
                     Mature                                                  Star
cgcauguucuucguuuaccuucuggaucuuguucgacagcacucccaacaacguuguuguuuuggaugauauagaugugcugucgaacaugauccagaaggugcugucgaacaacauacaaaaaggggucuacuguacguccggcccaucaugaucuucuucuucuucuucuucuucuucuuccugaaagaguaucaaacaugggguuucuguuuguuucuucuucuuuucuuuuuacaaguagauuua
.............................................................................uUcugucgaacaugauccaga.......................................................................................................................................................	1	1	JCK-1
.............................................................................ugcugucgaacaugCuccaga.......................................................................................................................................................	7	1	LT-2
.............................................................................ugcugucgCacaugauccaga.......................................................................................................................................................	3	1	LT-3
.............................................................................ugcugucgaacaugCuccaga.......................................................................................................................................................	4	1	JCK-3
.............................................................................ugcuguUgaacaugauccaga.......................................................................................................................................................	1	1	JCK-2
.............................................................................ugcugucgCacaugauccaga.......................................................................................................................................................	2	1	LCK-3
.............................................................................ugcuguAgaacaugauccaga.......................................................................................................................................................	1	1	LT-2
.............................................................................ugcugucgaacGugauccaga.......................................................................................................................................................	1	1	JCK-2
.............................................................................ugcugucgaacaugauccaAa.......................................................................................................................................................	1	1	JCK-2
.............................................................................ugcugucgGacaugauccaga.......................................................................................................................................................	7517	1	JCK-1
.............................................................................ugcugucgaacaugauccagC.......................................................................................................................................................	1	1	JCK-2
.............................................................................ugcugucgaacaugCuccaga.......................................................................................................................................................	3	1	JCK-2
.............................................................................ugcugucgaacaugaucAaga.......................................................................................................................................................	1	1	JCK-2
.............................................................................ugcugucgaacNugauccaga.......................................................................................................................................................	4	1	JCK-1
.............................................................................ugcugucgaacaugauccCga.......................................................................................................................................................	1	1	JCK-3
.............................................................................ugUugucgaacaugauccaga.......................................................................................................................................................	1	1	LT-3
.............................................................................ugcugucgaacaugauccaga.......................................................................................................................................................	118	0	JT-2
.............................................................................ugcugucgaacGugauccaga.......................................................................................................................................................	1	1	LCK-3
.............................................................................ugcugucgaCcaugauccaga.......................................................................................................................................................	3	1	JCK-1
.............................................................................ugcugucgaacaugCuccaga.......................................................................................................................................................	1	1	JCK-1
.............................................................................ugcugucgCacaugauccaga.......................................................................................................................................................	10	1	LT-2
.............................................................................ugcugucgaacauAauccaga.......................................................................................................................................................	3	1	LT-2
.............................................................................ugcugucgaacaugauccaga.......................................................................................................................................................	2228	0	LT-2
.............................................................................ugcugucgaacaugauccaga.......................................................................................................................................................	2141	0	LT-1
.............................................................................ugcugucgCacaugauccaga.......................................................................................................................................................	3	1	JCK-3
.............................................................................ugcugucgaCcaugauccaga.......................................................................................................................................................	3	1	LT-1
.............................................................................ugcugucgCacaugauccaga.......................................................................................................................................................	5	1	JCK-2
.............................................................................ugcugucgaGcaugauccaga.......................................................................................................................................................	1	1	JCK-2
.............................................................................uAcugucgaacaugauccaga.......................................................................................................................................................	1	1	JCK-2
.............................................................................ugcugucgCacaugauccaga.......................................................................................................................................................	3	1	JCK-1
.............................................................................ugcugucgaacaugauccaga.......................................................................................................................................................	1673	0	JCK-1
.............................................................................ugcugucgaGcaugauccaga.......................................................................................................................................................	1	1	JCK-3
.............................................................................uAcugucgaacaugauccaga.......................................................................................................................................................	1	1	LCK-3
.............................................................................uAcugucgaacaugauccaga.......................................................................................................................................................	1	1	JCK-3
.............................................................................ugcCgucgaacaugauccaga.......................................................................................................................................................	2	1	LT-1
.............................................................................ugcugCcgaacaugauccaga.......................................................................................................................................................	2	1	LT-3
.............................................................................ugNugucgaacaugauccaga.......................................................................................................................................................	7	1	LT-2
.............................................................................ugcugucgGacaugauccaga.......................................................................................................................................................	17	1	JT-2
.............................................................................ugcugucgaacaugauGcaga.......................................................................................................................................................	2	1	JCK-1
.............................................................................ugcugucgaacaugauccCga.......................................................................................................................................................	4	1	JCK-2
.............................................................................ugcugCcgaacaugauccaga.......................................................................................................................................................	1	1	JCK-2
.............................................................................ugcNgucgaacaugauccaga.......................................................................................................................................................	4	1	JCK-1
.............................................................................ugcugucgaacaugauccCga.......................................................................................................................................................	5	1	LT-2
.............................................................................uUcugucgaacaugauccaga.......................................................................................................................................................	1	1	JCK-2
.............................................................................ugAugucgaacaugauccaga.......................................................................................................................................................	1	1	LCK-3
.............................................................................ugcugucgGacaugauccaga.......................................................................................................................................................	9455	1	JCK-3
.............................................................................ugcugucgaacaugaucAaga.......................................................................................................................................................	3	1	LT-3
.............................................................................ugcugucgaacCugauccaga.......................................................................................................................................................	1	1	JCK-1
.............................................................................uNcugucgaacaugauccaga.......................................................................................................................................................	3	1	JCK-3
.............................................................................ugcugucgaacaugauccagC.......................................................................................................................................................	1	1	JT-3
.............................................................................uNcugucgaacaugauccaga.......................................................................................................................................................	4	1	JCK-2
.............................................................................uUcugucgaacaugauccaga.......................................................................................................................................................	2	1	LT-2
.............................................................................ugcugucgaacauAauccaga.......................................................................................................................................................	1	1	JCK-2
.............................................................................ugcugucgaCcaugauccaga.......................................................................................................................................................	5	1	LT-3
.............................................................................uAcugucgaacaugauccaga.......................................................................................................................................................	3	1	LT-1
.............................................................................ugcugucgaacGugauccaga.......................................................................................................................................................	1	1	JCK-3
.............................................................................ugcuguUgaacaugauccaga.......................................................................................................................................................	1	1	LT-1
.............................................................................ugcuguAgaacaugauccaga.......................................................................................................................................................	1	1	JCK-2
.............................................................................ugcugucgNacaugauccaga.......................................................................................................................................................	2	1	JCK-3
.............................................................................ugcugucgaacaugauccagC.......................................................................................................................................................	1	1	JCK-1
.............................................................................ugcugucgaacaugauccGga.......................................................................................................................................................	1	1	JCK-3
.............................................................................uAcugucgaacaugauccaga.......................................................................................................................................................	1	1	JCK-1
.............................................................................ugcugucgaacaugauccagC.......................................................................................................................................................	1	1	JT-1
.............................................................................ugcugucgaacaugauccaAa.......................................................................................................................................................	5	1	JCK-3
.............................................................................uAcugucgaacaugauccaga.......................................................................................................................................................	1	1	LT-2
.............................................................................ugcugCcgaacaugauccaga.......................................................................................................................................................	1	1	JCK-3
.............................................................................ugcugucgaacaugauccCga.......................................................................................................................................................	5	1	LT-3
.............................................................................uCcugucgaacaugauccaga.......................................................................................................................................................	1	1	LT-2
.............................................................................ugcugucgaacCugauccaga.......................................................................................................................................................	2	1	LT-2
.............................................................................ugcuguUgaacaugauccaga.......................................................................................................................................................	1	1	JCK-3
```

Mature Star

cgcauguucuucguuuaccuucuggaucuuguucgacagcacucccaacaacguuguuguuuuggaugauauagaugugcugucgaacaugauccagaaggugcugucgaacaacauacaaaaaggggucuacuguacguccggcccaucaugaucuucuucuucuucuucuucuucuucuuccugaaagaguaucaaacaugggguuucuguuuguuucuucuucuuuuucuuuuuacaaguagauuua

ugcugucgaacaugaucNaga	1	1	JCK-3
ugcugucgaCcaugauccaga	5	1	LT-2
ugcugucgaacaugauccagC	1	1	LT-3
ugcugucAaacaugauccaga	2	1	LT-2
ugcugucgCacaugauccaga	1	1	LT-1
ugcugucgaacauAauccaga	2	1	JCK-3
ugcugucgaacaugauccagU	5	1	JCK-1
ugcugucgUacaugauccaga	1	1	JCK-2
ugcugucgaacaugCuccaga	3	1	LT-3
ugcugucgGacaugauccaga	44	1	JT-1
ugcugucgaacaugauccaNa	4	1	JCK-1
ugcugucgGacaugauccagaa	14	1	JCK-3
ugcugucgGacaugauccagaa	12	1	JCK-2
ugcugucgaacaugauccagaa	1	0	JCK-1
ugcugucgaacaugauccagaU	3	1	JCK-3
ugcugucgaacaugauccagaU	4	1	LT-3
ugcugucgaacaugauccagaC	3	1	LT-1
ugcugucgaacaugauccagaa	3	0	LT-2
ugcugucgaacaugauccagaC	2	1	LT-3
ugcugucgaacaugauccagaC	3	1	JCK-2
ugcugucgaacaugauccagaa	2	0	JCK-2
ugcugucgaacaugauccagaC	8	1	LT-2
ugcugucgaCcaugauccagaa	1	1	LT-1
ugcugucgaacaugauccagaC	12	1	LCK-3
ugcugucgaacaugauccagaa	6	0	LT-1
ugcugucgaacaugauccagaU	1	1	LT-1
ugcugucgGacaugauccagaa	5	1	JCK-1
ugcugucgaacaugauccagaa	3	0	LT-3
ugcugucgaacaugauccagaU	3	1	LT-2
ugcugucgaacaugauccagaU	1	1	JCK-2
ugcugucgaacaugauccagaC	1	1	JCK-3
ugcugucgaacaugauccagaagg	1	0	JCK-3
ugcugucgaacaugauccagaagg	2	0	JCK-2
gcugucgaacaugauccag	6	0	LCK-3
gcugucgGacaugauccaga	5	1	JCK-3
gcugucgGacaugauccaga	9	1	JCK-2
gcugucgGacaugauccaga	2	1	JCK-1
cugucgaacaugauccag	1	0	LT-3
cugucgaacaugauccaga	8	0	LCK-3
cugucgaacCugauccaga	1	1	LT-2
cugucgaacaugauccaga	10	0	LT-3
cugucgaacaugauccaga	7	0	LT-1
cugucgCacaugauccaga	1	1	LT-1
cugucgaacaugauccagU	1	1	LT-2
cugucgaacaugauccaga	3	0	JCK-2
cugucgaacaugauccaga	21	0	LT-2
cugucgaacaugauccagaa	1	0	LT-2
cugucgaacaugauccagaU	7	1	LCK-3
cugucgaacaugauccagUa	2	1	LT-2
cugucgaacaugauccagaa	1	0	JCK-3
cugucgaacaugauccagaU	1	1	LT-1
cugucgaacaugauccagaa	4	0	LCK-3
cugucgaacaugauccagaa	3	0	LT-3
cugucgaacaugauccagaa	4	0	LT-1
cugucgaacaugauccagaa	2	0	JCK-2
cugucgCacaugauccagaag	1	1	LT-2
cugucgaacaugauccagaag	21	0	LCK-3
cugucgaacaAgauccagaag	9	1	LCK-3
cugucgaacaugauccagaag	10	0	LT-2
cugucgaacaugauccagaag	9	0	JCK-1
cugucgaacaugauccagaag	18	0	JCK-3
cugucgaacaugauccagaag	12	0	LT-1
cugucgaacaugauccagaaU	3	1	LT-1
cugucgCacaugauccagaag	1	1	LT-1
cugucgaacaugauccagaag	13	0	JCK-2
cugucgaacaugauccagaag	20	0	LT-3
cugucgaacaugauccagaaA	1	1	JCK-2
cugucgaacaugauccagaagA	3	1	JCK-2
cugucgaacaugauccagaagUu	1	1	LT-3
cugucgaacaugauccagaagUu	2	1	LT-2

Mature **Star**

```
cgcauguucuucguuuaccuucuggaucuuguucgacagcacucccaacaacguuguuguuuuggaugauauagaugugcugucgaacaugauccagaaggugcugucgaacaacauacaaaaaggggucuacuguacguccggcccaucaugaucuucuucuucuucuucuucuucuucuuccugaaagaguaucaaacaugggguuucuguuuguuucuucuucuuuuucuuuuuacaaguagauuua
................................................................................ugucgaacaugauccaga........................................................................................................................................................	2	0	LT-1
................................................................................ugucgaacaugauccaga........................................................................................................................................................	1	0	LT-3
................................................................................ugucgaacaugauccagaagg.....................................................................................................................................................	5	0	LT-1
................................................................................ugucgaacaugauccagaagg.....................................................................................................................................................	4	0	LT-3
................................................................................ugucgaacaugauccagaagg.....................................................................................................................................................	2	0	JCK-2
................................................................................ugucgaacaugauccagaagg.....................................................................................................................................................	2	0	JCK-3
................................................................................ugucgaacaugauccagaagg.....................................................................................................................................................	6	0	LT-2
..................................................................................ucgaacaugauccagaag......................................................................................................................................................	1	0	LT-1
..................................................................................ucgaacaugauccagaagg.....................................................................................................................................................	1	0	JCK-1
..................................................................................ucgaacaugauccagaaggu....................................................................................................................................................	1	0	LT-2
..................................................................................ucgaacaugauccagaaggug...................................................................................................................................................	15	0	LT-3
..................................................................................ucgaacaugauccagaaggug...................................................................................................................................................	23	0	LCK-2
..................................................................................ucgaacaugauccagaaggug...................................................................................................................................................	1	0	JCK-1
..................................................................................ucgaacaugauccagaaggug...................................................................................................................................................	9	0	LT-2
..................................................................................ucgaacaugauccagaaggug...................................................................................................................................................	2	0	JCK-2
..................................................................................ucgaacaugauccagaaggug...................................................................................................................................................	16	0	LT-1
..................................................................................ucgaacaugauccagaaggug...................................................................................................................................................	27	0	LCK-1
..................................................................................ucgaacaugauccagaagguA...................................................................................................................................................	1	1	LT-1
...................................................................................cgaacaugauccagaaggu....................................................................................................................................................	1	0	LT-2
...................................................................................cgaacaugauccagaaggu....................................................................................................................................................	2	0	LT-3
...................................................................................cgaacCugauccagaaggu....................................................................................................................................................	1	1	LT-3
...................................................................................cgaacaugauccagaaggug...................................................................................................................................................	2	0	LT-3
...................................................................................cgaacaugauccagaaggug...................................................................................................................................................	3	0	LT-1
...................................................................................cgaacaugauccagaaggug...................................................................................................................................................	1	0	JCK-2
...................................................................................cgaacaugauccagaaggugc..................................................................................................................................................	3	0	JCK-1
...................................................................................cgaacaugauccagaaggugc..................................................................................................................................................	6	0	LT-1
...................................................................................cgaacaugauccagaaggugc..................................................................................................................................................	4	0	JCK-3
...................................................................................cgaacaugauccagaaggugc..................................................................................................................................................	3	0	LT-3
...................................................................................cgaacaugauccagaaggugc..................................................................................................................................................	6	0	JCK-2
.....................................................................................aacaugauccagaaggugcugucgaaca.........................................................................................................................................	2	0	JCK-3
......................................................................................acaugauccagaaggugcugucg.............................................................................................................................................	1	0	LT-3
......................................................................................acaAgauccagaaggugcugucga............................................................................................................................................	2	1	JCK-1
......................................................................................acaAgauccagaaggugcugucga............................................................................................................................................	2	1	JCK-3
......................................................................................acaAgauccagaaggugcugucga............................................................................................................................................	1	1	JCK-2
......................................................................................acaugauccagaaggugcugucgaa...........................................................................................................................................	1	0	JCK-1
......................................................................................acaugauccagaaggugcugucgaac..........................................................................................................................................	2	0	JCK-1
......................................................................................acaugauccagaaggugcugucgaac..........................................................................................................................................	1	0	JCK-2
......................................................................................acaugauccagaaggugcugucgaac..........................................................................................................................................	1	0	JCK-3
.......................................................................................caugauccagaaggugcugucgaaca.........................................................................................................................................	1	0	LT-1
............................................................................................uccagaaggugcugucgaaca.........................................................................................................................................	1	0	LT-3
............................................................................................uccagaaggugcugucgaaca.........................................................................................................................................	2	0	LT-1
............................................................................................uccagaaggugcugucgaaca.........................................................................................................................................	2	0	LT-2
............................................................................................uccagaaggugcugucgaacaa........................................................................................................................................	3	0	LT-1
............................................................................................uccagaaggugcugucgaacaa........................................................................................................................................	1	0	LT-3
............................................................................................uccagaaggugcugucgaacaac.......................................................................................................................................	5	0	LCK-3
............................................................................................uccagaaggugcugucgaacaac.......................................................................................................................................	12	0	JCK-2
............................................................................................uccagaaggugcugucgaacaac.......................................................................................................................................	8	0	LT-3
............................................................................................uccagaaggugcugucgaacaac.......................................................................................................................................	2	0	LT-1
............................................................................................uccagaaggugcugucgaacaac.......................................................................................................................................	15	0	JCK-3
............................................................................................uccagaaggugcugucgaacaac.......................................................................................................................................	4	0	JCK-1
............................................................................................uccagaaggugcugucgaacaac.......................................................................................................................................	4	0	LT-2
............................................................................................uccagaaggugcugucgaacaacU......................................................................................................................................	1	1	LT-3
............................................................................................uccagaaggugcugucgaacaacN......................................................................................................................................	1	1	LT-2
............................................................................................uccagaaggugcugucgaacaacU......................................................................................................................................	1	1	JCK-2
............................................................................................uccagaaggugcugucgaacaaca......................................................................................................................................	2	0	LT-2
............................................................................................uccagaaggugcugucgaacaaca......................................................................................................................................	2	0	JCK-3
............................................................................................uccagaaggugcugucgaacaacU......................................................................................................................................	6	1	LCK-3
............................................................................................uccagaaggugcugucgaacaaca......................................................................................................................................	3	0	LT-3
............................................................................................uccagaaggugcugucgaacaacU......................................................................................................................................	2	1	LT-1
...............................................................................................agaaggugcugucgaaca.........................................................................................................................................	1	0	LT-3
...............................................................................................agaUggugcugucgaacaaca......................................................................................................................................	1	1	JCK-3
...............................................................................................agaUggugcugucgaacaaca......................................................................................................................................	1	1	JCK-2
...............................................................................................agaaggugcugucgaacaaca......................................................................................................................................	4	0	LCK-3
...............................................................................................agaaggugcugucgaacaacau.....................................................................................................................................	1	0	LT-1
...............................................................................................agaaggugcugucgaacaacau.....................................................................................................................................	1	0	LT-3
...............................................................................................agaaggugcugucgaacaacaua....................................................................................................................................	2	0	LT-2
...............................................................................................agaaggugcugucgaacaacaua....................................................................................................................................	2	0	LT-1
................................................................................................gaaggugcugucgaacaacauacaaaaag.............................................................................................................................	1	0	JCK-1
....................................................................................................gugcugucgaacaaGaua....................................................................................................................................	2	1	LCK-3
....................................................................................................gugcugucgaacaaGaua....................................................................................................................................	1	1	JCK-2
```

Mature Star				
cgcauguucuucguuuaccuucuggaucuuguucgacagcacucccaacaacguuguuguuuuggaugauauagaugugcugucgaacaugauccagaaggugcugucgaacaacauacaaaaaggggucuacuguacguccggcccaucaugaucuucuucuucuucuucuucuucuucuuccugaaagaguaucaaacaugggguuucuguuuguuucuucuucuuuucuuuuuacaaguagauuua				
....uuccugaaagaguaucaaac...		5	0	LT-2
